# Sequencing of Supernumerary Chromosomes of Red Fox and Raccoon Dog Confirms a Non-Random Gene Acquisition by B Chromosomes

**DOI:** 10.3390/genes9080405

**Published:** 2018-08-10

**Authors:** Alexey I. Makunin, Svetlana A. Romanenko, Violetta R. Beklemisheva, Polina L. Perelman, Anna S. Druzhkova, Kristina O. Petrova, Dmitry Yu. Prokopov, Ekaterina N. Chernyaeva, Jennifer L. Johnson, Anna V. Kukekova, Fengtang Yang, Malcolm A. Ferguson-Smith, Alexander S. Graphodatsky, Vladimir A. Trifonov

**Affiliations:** 1 Institute of Molecular and Cellular Biology Siberian Branch of the Russian Academy of Sciences, 630090 Novosibirsk, Russia; rosa@mcb.nsc.ru (S.A.R.); bekl@mcb.nsc.ru (V.R.B.); polina.perelman@gmail.com (P.L.P.); rada@mcb.nsc.ru (A.S.D.); dprokopov@mcb.nsc.ru (D.Y.P.); graf@mcb.nsc.ru (A.S.G.); vlad@mcb.nsc.ru (V.A.T.); 2 Novosibirsk State University, 630090 Novosibirsk, Russia; petrova@mcb.nsc.ru; 3 Theodosius Dobzhansky Center for Genome Bioinformatics, Saint-Petersburg State University, 199004 Saint-Petersburg, Russia; echernya@gmail.com; 4 Department of Animal Sciences, University of Illinois at Urbana-Champaign, Urbana, IL 61801, USA; jjohnso@illinois.edu (J.L.J.); avk@illinois.edu (A.V.K.); 5 Wellcome Sanger Institute, Wellcome Genome Campus, Hinxton, Cambridge CB10 1SA, UK; fy1@sanger.ac.uk; 6 Cambridge Resource Centre for Comparative Genomics, Department of Veterinary Medicine, Cambridge University, Cambridge CB3 0ES, UK; maf12@cam.ac.uk

**Keywords:** supernumerary chromosomes, karyotype evolution, genome instability

## Abstract

B chromosomes (Bs) represent a variable addition to the main karyotype in some lineages of animals and plants. Bs accumulate through non-Mendelian inheritance and become widespread in populations. Despite the presence of multiple genes, most Bs lack specific phenotypic effects, although their influence on host genome epigenetic status and gene expression are recorded. Previously, using sequencing of isolated Bs of ruminants and rodents, we demonstrated that Bs originate as segmental duplications of specific genomic regions, and subsequently experience pseudogenization and repeat accumulation. Here, we used a similar approach to characterize Bs of the red fox (*Vulpes vulpes* L.) and the Chinese raccoon dog (*Nyctereutes procyonoides procyonoides* Gray). We confirm the previous findings of the *KIT* gene on Bs of both species, but demostrate an independent origin of Bs in these species, with two reused regions. Comparison of gene ensembles in Bs of canids, ruminants, and rodents once again indicates enrichment with cell-cycle genes, development-related genes, and genes functioning in the neuron synapse. The presence of B-chromosomal copies of genes involved in cell-cycle regulation and tissue differentiation may indicate importance of these genes for B chromosome establishment.

## 1. Introduction

B chromosomes (or Bs) were first described over a century ago [1] and since then they have been found in a multitude of animal and plant species. The name reflects the fact that the Bs represent a variable addition to the normal (or A) chromosome set. Unlike other types of supernumerary chromosomes, such as marker chromosomes [2], double minutes [3], and circular extrachromosomal DNA [4], Bs are widespread in populations and generally lack specific phenotypic effects. They often demonstrate a higher than Mendelian inheritance rate, resulting from non-random behavior during cell division: Accumulation in gametes relative to somatic cells or in ovaries relative to polar bodies. This phenomenon is known as the B chromosome drive [5,6].

In most species studied so far, Bs originated through ectopic segmental duplications of main genome chromosomal fragments, although cases involving interspecific hybridization [7] or polyploidization [8] are described. Subsequently, Bs undergo internal amplification, accumulation of additional DNA sequences—both unique and repetitive. Eventually, Bs degrade and disappear from the genome. Currently there are no documented cases of Bs older than several million years [9,10,11].

Several studies identified genes on Bs of plants, insects, and vertebrates, and for all of these lineages records of the transcribed B-chromosomal gene copies exist (reviewed in Reference [12,13,14]). B chromosomes were found to contain genes associated with cell division and tissue differentiation, suggesting their role in the Bs drive [10,15,16,17].

The first report of a protein coding gene on Bs, namely protooncogene *KIT*, was made for the red fox and raccoon dog [18]. Subsequent study refined the duplication breakpoints [19]. As a result of high-density bacterial artificial chromosome (BAC)-clone mapping of fox and raccoon dog genomes, the number of B-chromosomal regions increased to seven in the red fox and to five in the raccoon dog [20]. Since then, development of high-throughput sequencing and associated analytical methods allowed us to directly access the genetic content of Bs in mammals using samples of chromosomes isolated by flow sorting and microdissection [21,22].

Here, we utilize this approach to access the genetic content of Bs in the red fox and Chinese raccoon dog based on flow-sorted and microdissected chromosome sequencing. For the red fox, we supplement Bs sequencing data with copy number estimation based on the whole-genome sequencing data. We also combine Bs gene content for six species of mammals analyzed so far [21,22] to identify unifying features of genes involved in Bs formation.

## 2. Materials and Methods

Chromosomes were isolated from established primary fibroblast cell cultures using chromosome flow sorting [23,24] of several hundred chromosome copies, or microdissection [18,25,26] of single chromosome copies. For both methods, whole-chromosome amplification was performed either with degenerate oligonucleotide-primed polymerase chain reaction (DOP-PCR) [27] using 6-MW primer (5′-CCGACTCGAGNNNNNNATGTGG-3′) or with whole-genome amplification WGA1 kit (Sigma-Aldrich, Saint Louis, MO, USA) depending on the sample (Table S1). For newly acquired microdissected samples, the specificity of amplified chromosome DNA was checked with fluorescence in situ hybridization to metaphase chromosomes of the corresponding species (Figures S1 and S2) [18,23,24,25].

Samples were prepared for sequencing with Nextera or TruSeq v.3 library preparation kits (Illumina, San Diego, CA, USA). Paired-end sequencing was performed using Illumina MiSeq (Table S1). Sequencing data are available from NCBI short read archive (SRA) under accession PRJNA477942.

Genomic regions of chromosomes were identified using dopseq_pipeline of DOPseq_analyzer v.1.0 [21], which includes primer and adapter trimming with cutadapt v.1.8.3 [28], alignment to the dog (CanFam3.1) genome with BWA-MEM v.0.7.15 [29], removal of human contamination by comparative alignment to human (hg19) genome, filtering of the alignment (min_mapq = 20, min_len = 20), and genome classification based on mean distance between consecutive merged mapped read positions, which is expected to be lower for regions present on the sampled chromosome (see Table S2 for statistics). Only chromosomes of the dog genome assembly were considered at this point, while unassigned scaffolds were discarded. Automated target region detection was complemented by visual inspection of rainfall-style plots representing the distribution of distances between mapped reads along the reference chromosomes (Supplementary File S2).

Copy-number analysis was performed using genomic reads (PRJNA378561) generated during the fox genome assembly project [30]. We established a fibroblast cell culture from this individual and estimated the Bs number to be stable and equal to three. We microdissected all three Bs from a single metaphase plate. The resulting DNA samples were amplified, sequenced and used in this study as VVUB3, 4, and 6. Fox genomic reads were aligned to CanFam3.1 genome with BWA-MEM, and copy number estimation was performed with CNVnator v.3.3.0 [31]. The changes in read coverage corresponding to duplications and deletions were estimated with a sliding window size of 1000 bp, as a lower window size (200 bp) revealed a high number of false positive deletions resulting from uneven coverage for between-species read mapping. B chromosomal copy number was estimated from normalized read depth reported by CNVnator (Table S3).

As a part of comparative gene content study, we re-analyzed Siberian roe deer (*Capreolus pygargus* Pall.) and grey brocket deer (*Mazama gouazoubira* G. Fischer) Bs sequencing data [21] with dopseq_pipeline, using cattle reference genome UMD3.1 instead of Baylor Btau_4.6.1 and BWA-MEM instead of Bowtie2 with “very-sensitive-local” profile, as in the original publication [21]. Other parameters were set as described above for canid chromosomes.

Sets of genes found in Bs of six mammalian species were obtained from the Ensembl Genes 92 [32] database using R biomaRt package [33]. First, B-chromosomal gene sets were obtained for the original reference genomes: dog for red fox and Chinese raccoon dog, cattle for Siberian roe deer and grey brocket deer, mouse for field mice *Apodemus flavicollis* Melchior and *Apodemus peninsulae* Thomas. Then, information on gene homology was added, so that every gene was supplemented (if possible) with identifiers in human, mouse, dog, and cattle genomes (Supplementary File S3). Functional enrichment analysis was performed using DAVID GO v.6.8 [34,35] for the selections of genes representing all or one-to-one (i.e., single copy) orthologs in the human genome (Supplementary File S4). Functional clustering was performed only for categories that annotated at least 80% of genes in the gene sets. Default background datasets of human genes were used for each reference.

## 3. Results

### 3.1. Sequencing of Fox and Raccoon Dog B Chromosomes and Autosomes

To ascertain the adequacy of our approach for chromosomal region detection we included samples of autosomes: flow-sorted Chinese raccoon dog (*Nyctereutes procyonoides procyonoides* Gray, NPP) chromosome 6 (NPP6) and microdissected red fox (*Vulpes vulpes* L., VVU) chromosome 3 (VVU3). In both cases, detected regions are in good agreement with comparative cytogenetics data [20,23,24]: NPP6 is homologous to the entire dog (*Canis lupus familiaris* L., CFA) chromosome 3 (CFA3) and the distal portion of CFA13; VVU3 is homologous to entire chromosomes CFA6, CFA34, and CFA36. For NPP6, an additional 160 kbp region of CFA16 was found, suggesting a putative duplication or translocation. This result indicates the higher resolution of our method compared to comparative cytogenetics, but requires further validation. Slight depletion of reads in some genomic regions was observed on autosomes of both species: In a region of NPP6 corresponding to CFA3:56.4-62.8 Mbp and in a region of VVU3 corresponding to CFA6:55.8 Mbp up to chromosome end for VVU3 (Supplementary File S2). These changes were insufficient to be treated as deletions, but reflected a trend of under-representation of certain regions in isolated chromosome sequencing data [21].

Four samples of fox Bs were sequenced, including one sorted (VVUB2) [23] and three microdissected from a single metaphase plate (VVUB3, 5 and 6). Comparison of sequenced Bs to the dog genome sequence revealed 14 regions comprising 7.7 Mbp (Table 1). Three samples, including both sorted and microdissected Bs (VVUB2, 3 and 5), were in perfect agreement. A reduced set of regions was identified in the sample VVUB6. The seven regions previously detected in fox Bs by BAC clone mapping [20] were recovered successfully. Significant deletions lacking read coverage were observed in two regions: CFA13:34 Mbp and CFA22:24-25 Mbp.

For the Chinese raccoon dog, a total of eight samples of Bs were analyzed: One sorted (NPPB1), and others microdissected (NPPB2-8) (Table 2). Here, the highest number of regions (27, total size 8.5 Mbp) was recovered from the sorted sample only, while microdissected samples demonstrated only a subset of these regions—in contrast to fox Bs, where both chromosome isolation methods resulted in similar sets of regions. We recovered only three of five regions previously identified on Chinese raccoon dog Bs by BAC clone mapping [20]. The region corresponding to CFA13:34 Mbp claimed to be present on both fox and raccoon dog Bs was discovered only in fox Bs, while the region on CFA29:4 Mbp (specific to Chinese raccoon dog) was not identified in any samples. Due to the larger size of raccoon dog Bs, in addition to whole-chromosome probes (NPPB2 and 3), we were able to dissect proximal (NPPB5 and 6), middle (NPPB7), and distal (NPPB4 and 8) portions of the B, hoping to recover gene order on the B. However, this approach had only limited success: Differences between same-portion samples (NPPB5 vs. 6, 4 vs. 8, see Table 2) were higher than between samples of different chromosomal portions from the same experiment (NPPB4 vs. 5, 6 vs. 7 vs. 8), and many regions were present throughout the whole B. This finding is in line with previous BAC-clone mapping results, which yielded repetitive locations of several genes throughout the NPPB [20].

**Figure 1:**
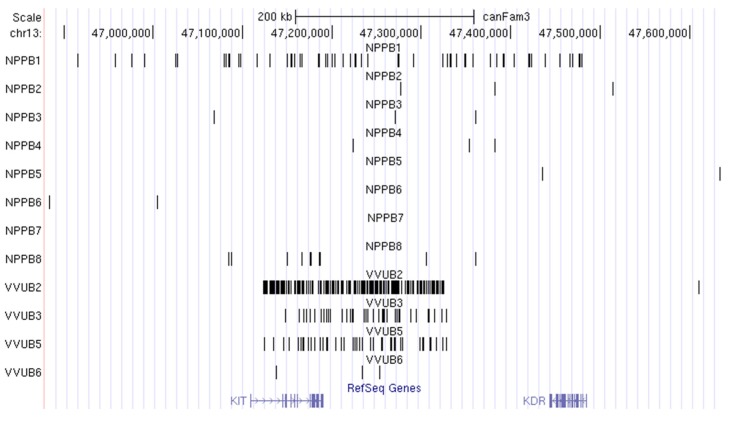
The region encompassing protooncogene *KIT* is present on B chromosomes (Bs) of the fox (VVUB2, 3, 5 and 6) and raccoon dog (NPPB1-8) visualized in UCSC genome browser (http://genome.ucsc.edu/). Coordinates are given for the dog (CanFam3.1) genome.

We found two overlapping regions on Bs of the fox and raccoon dog: CFA13:47 Mbp with *KIT* protooncogene (Figure 1), which was the first gene to be discovered in Bs of mammals [18], and CFA32:13-15 Mbp, with no genes in the subregion present in both species. A similar region reuse pattern involving regions without any genes was previously observed for Bs in two field mouse species [22]. Several regions identified on Bs of the red fox and raccoon dog are located in close proximity in the dog genome, e.g., CFA15:53-54 Mbp in fox Bs and CFA15:58 Mbp in raccoon dog Bs, CFA19:41-44 Mbp (VVUB) and CFA19:38-39 Mbp (NPPB), CFA29:23 Mbp (VVUB) and CFA29:28-29 Mbp (NPPB).

### 3.2. Fox Whole-Genome Sequencing Data

Whole-genome sequencing (WGS) allows the discovery of B-chromosomal regions, using coverage-based copy number analysis of individuals with B-chromosomes (B+) and comparison to individuals without Bs (B−) [10]. The regions with increased copy number only in the B+ genome are treated as present on Bs.

Here, we took advantage of the WGS data available for the fox individual whose fibroblast cell cultures were used for isolation of Bs VVUB3, 5 and 6. We aligned its reads to the dog genome and called copy number variants, anticipating finding a higher coverage in B-specific regions. We were able to avoid the WGS for the B− individual by focusing on the regions predicted with isolated B chromosome sequencing. The overall agreement between whole-genome copy number and isolated chromosome sequencing results was striking—all 14 regions were consistently recovered by both methods (Table S3). The median difference between breakpoint coordinates predicted by the two methods was about 700 bp, or less than one window size (1000 bp)—the limit of copy number calling resolution. Compared to the isolated chromosome sequencing, the copy-number dataset included multiple internal deletions, which presumably reflect unevenly reduced mapping efficiency for the cross-species read alignment. As a result, the total size of the B chromosomal regions detected by copy number analysis (6.2 Mbp, Table S3) was still significantly lower than for isolated chromosome sequencing (7.4 Mbp).

**Figure 2:**
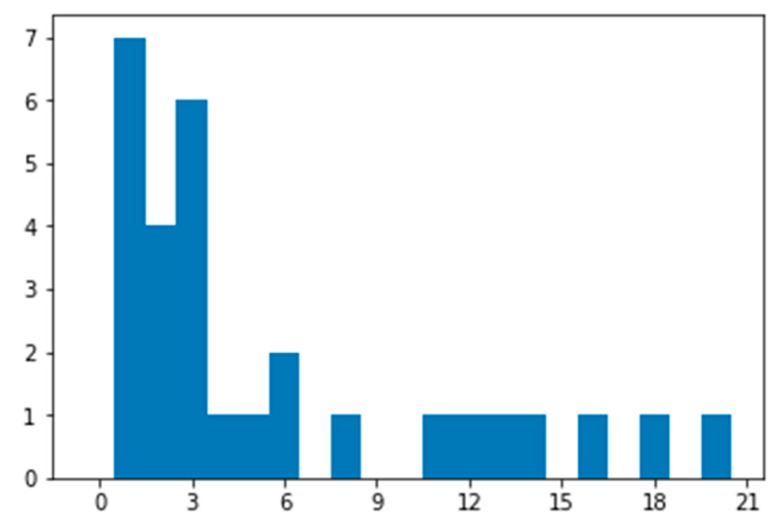
Numbers of additional copies for B chromosomal regions (identified by isolated chromosome sequencing) estimated based on whole-genome sequencing of the red fox individual with three Bs. Region parts with different copy numbers counted separately. Regions with copy number below three were lost from some of Bs, while regions with copy number above three were amplified within Bs. X—number of additional copies, Y—counts of regions.

Estimates of normalized read depth allowed us to calculate the copy number for each of the 14 regions present on Bs and compare it to the number of Bs of the sampled individual (*n* = 3, Figure 2). In agreement with BAC clone mapping results [18,19,20], some of the regions were amplified, e.g., six copies of *KIT* region (CFA13:47 Mbp) were present on Bs, which implies two copies per B chromosome. The copy number varied not only between the regions but also within some regions, suggesting a partial amplification of these regions in Bs. The most striking example was observed in the region corresponding to CFA31:2–4 Mbp; the number of additional copies for different parts of this region varied from zero (putative deletion) to 20 (presumably seven copies per B chromosome). Existence of copy numbers lower than three together with the absence of these regions from chromosome VVUB6 suggest that B heterogeneity within an individual exists (Table 1, Table S3). Taken together, these observations suggest that a complex mixture of duplications and deletions occurred since the origin of Bs in red fox, which resulted in significant heterogeneity among Bs—even within a single individual.

### 3.3. Genetic Content of B Chromosomes in Deer Revisited

B chromosomes of two deer species, Siberian roe deer (*C. pygargus* Pall., CPYB) and grey brocket deer (*M. gouazoubira* G. Fischer, MGOB) were previously sequenced and analyzed using an earlier version of the chromosome region identification pipeline [21]. Here, we repeatedly identified target region using another version of the cattle (*Bos taurus* L., BTA) reference genome assembly (UMD3.1) and a more sensitive alignment algorithm. For both samples, this resulted in a significant increase in B region numbers.

In roe deer Bs, five regions were identified in addition to the two discovered previously (Table S4). The largest added region corresponded to BTAX:148 Mbp (292 kbp in size). It is interesting that the alignment to UMD3.1 assembly with Bowtie2 also revealed this region. Thus, this region became detectable due to the significant improvement of the sex chromosome assembly, rather than due to increased aligner sensitivity. The total size of B chromosomal regions increased from 1.96 Mbp to 2.36 Mbp.

In grey brocket deer, four regions were detected in addition to 25 identified previously (Table S5), and the size was increased from 9.31 Mbp to 10.46 Mbp. Among the newly discovered regions was the retrogene, *RASA1*, for which only exons were located in Bs. Similarly processed retrogenes were previously reported for Bs of fish [10].

In both species, false positive signals arose at stretches of telomeric (BTA20:72 Mbp) and centromeric (BTSAT4 at BTA19:0.4 Mbp, BTA:61 Mbp) repeats. The telomeric repeats are universal among mammals, and the observation of the same centromeric repeat in deer and cattle is in line with the previous report [36].

### 3.4. Comparison of B Chromosome Gene Content in Six Species of Mammals

To identify common features of Bs in various lineages of mammals, we compared genetic content of Bs across six species: red fox (VVUB), Chinese raccoon dog (NPPB), Siberian roe deer (CPYB), grey brocket deer (MGOB), and two field mouse species, yellow-necked mouse (*A. flavicollis* Melchior, AFLB) and Korean field mouse (*A. peninsulae* Thomas, APEB) [22]. For each species, we extracted Ensembl gene predictions overlapping the B chromosomal regions. The number of genes identified with such a method was higher than in both original studies, as other gene types, apart from protein-coding ones, were included in the analysis. Data on homologous human genes were also added from the Ensembl database (Table 3, Supplementary File S3).

Next, we ran functional annotation and clustering for human one-to-one homologs using the DAVID GO web interface (Table 4, Supplementary File S4). Unexpectedly, we found the top cluster to be associated with neuron synapses and cell junctions (enrichment score 2.15). Genes from this cluster were present on Bs of all species, except for the Siberian roe deer.

The cluster of cell division machinery and cell cycle control functions (enrichment score 1.26) included genes found in Bs of the grey brocket deer, both mouse species, and the red fox. A related cluster was associated with microtubules and centrosomes (enrichment score 0.94).

The B chromosomal gene set was also highly enriched with genes bound by developmental transcription factors (enrichment score 2.09). Still, genes involved in cell differentiation and proliferation did not form a significant cluster, although genes involved in these processes were found on Bs in all species studied. Although not automatically classified, *TNNI3K*, the first gene identified in Siberian roe deer Bs and the only confirmed example of transcribed B chromosomal gene in mammals [37], was included in this list based on the report of its role in cardiomyocyte differentiation from embryonic stem cells [38].

Multiple clusters were associated with specific intracellular processes: A cluster of genes found in all sampled Bs was associated with protein phosphorylation (enrichment score 1.14). The clusters with related protein domains included PH-like or Pleckstrin homology-like (phosphatidylinositol binding, enrichment score 0.97), SH or Src homology (phosphotyrosine binding, enrichment score 0.83), C2 domain (potentially phospholipid binding, found in Ca^2+^-dependent channels, enrichment score 1.57), and immunoglobulin-like domains (enrichment score 1.59).

In line with previous observations [18,20,39], genes involved in cancer development were represented on Bs of four studied species: red fox (*KIT*), Chinese raccoon dog (*ENPP1*, *GNAS*, *HMGCR*, *KDR*, *RET*), brocket deer (*BCL6*, *RASA1*, *RET*), and Korean field mouse (*JAK3*). Still, the enrichment with this function was not significant (score 0.09, *p*-value > 0.05).

## 4. Discussion

Canidae is the only family of carnivores with species bearing Bs. Bs of the red fox (*V. vulpes*) and raccoon dog (*N. procyonoides*) are the most studied, while only brief descriptions were made for Bs of the maned wolf (*Chrysocyon brachyurus*) [40], short-eared dog (*Atelocynus microtis*) [41], Bengal fox (*V. bengalensis*) [42], and pale fox (*V. pallida*) [43].

Dot-like Bs of red fox were one of the first to be described among mammals [44,45]. During the famous experiment on fox domestication [46], Bs were found to be replicating late [47] and somatically mosaic with accumulation in gametes [48]. In meiosis, Bs form uni-, bi- and trivalents and occasionally associate (but do not conjugate) with sex chromosomes [49]. Furthermore, meiotic recombination between Bs was hypothesized based on mismatch repair protein visualization [50]. Fox Bs are characterized by lower DNA methylation [51] and central location in interphase nuclei [52].

The revolutionary finding of the first unique protein-coding gene, namely protooncogene *KIT*, on Bs of mammals was made with the fox [18]. Further studies provided additional data on breakpoint margins [19] and identified six additional regions apart from the *KIT* region on red fox Bs [20]. The current data entirely confirmed these findings and allowed the identification of seven additional regions. Using a combination of isolated chromosome and whole-genome sequencing data, we demonstrated a complex pattern of DNA amplification on Bs: Many regions are present in single copy, while some are duplicated, amplified to a higher copy number, or deleted—partially or completely. Both methods suggest that one of three Bs in the studied individual (VVUB6) was partially degenerated in comparison to the other two (VVUB3 and 5).

In raccoon dogs, Bs were described in three subspecies: Chinese (*N. p. procyonoides*, NPP) [53], Japanese (*N. p. viverrinus*, NPV) [54], and Korean (*N. p. koreensis*) [55]. Multiple studies were made for Chinese and Japanese subspecies, which differ in the number of Robertsonian translocations and by B morphology, and supposedly represent independent species [56]. In contrast to the fox Bs, which are the smallest karyotype elements comparable only to Y chromosomes, the raccoon dog Bs are similar in size to medium (NPP) or small (NPV) autosomes [24,56]. In both subspecies, somatic mosaicism and variable B chromosome morphology were recorded [57,58,59,60]. NPP Bs form bi- and multivalents in meiosis [61], have higher level of DNA methylation [51] and are located at the periphery of the interphase nucleus [52]. Interstitial telomeric sites are found along the arms of Bs of both subspecies [62]. 28S rRNA gene clusters were found on NPP Bs [59].

Protooncogene *KIT* was discovered in both NPP and NPV Bs [18]. In NPP, B chromosomal copies of *KIT* were found to be enriched with sequence variants, but not transcribed [63]. Using PCR mapping it was shown that the *KIT* region in NPP Bs also includes the 5′ part of neighboring *KDR* gene [19]. Further high-density BAC clone mapping experiment identified four additional regions on NPP Bs [20], but the current data did not detect two of these regions: CFA13:34 Mbp and CFA29:41 Mbp. Both of these demonstrated only one pair of BAC clone hybridization signals per three Bs in the individual studied, and thus might represent either variable additional regions, as described in *A. flavicollis* Bs [22], or mapping artifacts, e.g., due to specific repeat accumulation. For the remaining three regions, the current dataset agrees well with BAC clone mapping. For example, *KIT* region location in the distal subarm is confirmed by both methods. Extensive amplification of genes found on NPP Bs is supported by three facts: recurrent identification of the same regions in different B subarms; significant increase of B total size (~50–100 Mbp as suggested by similar autosome sizes) in comparison to 8.5 Mbp unique chromosomal regions identified; earlier evidence of repetitive BAC clone localizations [18,19,20].

A relationship between Bs of raccoon dog subspecies remains unknown. The onset of Bs in the common ancestor could have predated the set of Robertsonian translocations observed in the Japanese raccoon dog. However, the only unique gene shown to be present in NPV Bs is *KIT*. This is the most frequently reused B chromosomal gene, which makes an independent origin of Bs also possible. Sequencing of the Japanese raccoon dog Bs could help elucidate a common or independent origin of Bs in NPP and NPV.

Genetic content of Bs is far from random. Previous studies suggested that Bs often harbor genes involved in cell division and early development. Two common B drive mechanisms found in animals imply interventions into these two processes: Directed (non)disjunction towards ovaries in female meiosis and mitotic accumulation in the germ line, as opposed to somatic tissues. Genes related to cell cycle and tissue differentiation were reported in non-mammalian lineages: morphogene *ihhb* (Indian hedgehog b) on Bs of cichlid *Lithochromis rubripinnis* [15]; several cell division associated genes in another cichlid *Astatotilapia latifiscata* [10]; a set of expressed pseudogenes including kinesins, argonaute and other regulatory genes in rye (*Secale cereale*) [16,64]; five genes related to cell division (*CIP2A*, *KIF20A*, *CKAP2*, *CAP-G* and *MYCB2*) in a grasshopper *Eyprepocnemis plorans* [17].

Similar tendencies were observed in mammals, both in terms of gene function enrichment and reuses (genes encoding cell-cycle related signaling protein kinases—*KIT*, *RET* and *VRK1*, as well as splicing regulatory *KHDRBS3*). The only species with Bs seemingly lacking genes related to cell cycle or early development is the Siberian roe deer, in which the first gene described and shown to be transcribed, *TNNI3K*, [37] has been recently associated with cardiovascular cell differentiation [38].

Neuron synapse and signaling represents another interesting function highly enriched in mammalian Bs. These genes can be found on Bs of all species studied, except for the roe deer. Examples of genes from this category also occur in Bs of cichlid *Astatotilapia latifiscata* [10]. This functional category remains quite unexplained by the current model of B chromosome evolution.

Five single copy genes were found to be reused in the Bs of different species. Protooncogene *KIT* was the first gene identified on the Bs in mammals, and it is currently found in Bs of three species: red fox, Chinese raccoon dog, and grey brocket deer. It encodes a protein kinase controlling the cell cycle, and plays an important role in development. Another protooncogene encoding protein kinase, *RET*, is found on Bs of Chinese raccoon dog and grey brocket deer. A third previously known gene reuse example is *VRK1*, a gene which also encodes a protein kinase involved in cell cycle control. This gene is found in Bs of two field mice species, which most likely acquired the supernumerary chromosomes independently [22].

Two novel examples of gene reuse were identified in this study:

- *KHDRBS3* (as known as *T-STAR* and *SLM-2*) is found on Bs of the grey brocket deer and the Korean field mouse. It encodes an RNA-binding signal transduction protein involved in alternative splicing regulation expressed in the brain and gonads. Mutations in this gene are associated with a neurological disorder (child absence epilepsy [65]), and affect the progression of various cancers.
- *MYOF* was found on Bs in the Chinese raccoon dog and the yellow-necked mouse. It encodes myoferin, a plasma-associated protein involved in Ca^2+^-channel formation, important in myoblast functioning [66] and involved in EGF-induced cell migration in breast cancer [67].

Two factors seemingly affect the onset of Bs. Genomic instability context, that is, propensity to chromosome rearrangements, is important, as it provides the source material for B chromosome formation. However, the fixation of Bs within a population may in fact depend on their own genes. Here, we revealed patterns of functional preferences and gene reuse in six mammalian species. These trends are, in general, compatible with the B chromosomal gene findings in other lineages. Further studies are needed to understand the exact molecular mechanisms that Bs used to “hack” cell division and differentiation processes while escaping from selection pressure.

## Figures and Tables

**Table 1:** Genomic regions identified in red fox (*Vulpes vulpes* L., VVU) B chromosomes (Bs). Region coordinates are given according to the dog (CanFam3.1, CFA) genome assembly. VVUB2—flow sorted B sample, VVUB3, 5 and 6—three Bs microdissected from a single metaphase plate, BAC—bacterial artificial chromosome (BAC) clone mapping data from Reference [20].

Region	VVUB2	VVUB3	VVUB5	VVUB6	BAC
CFA5:70796855-70973839	+	+	+		
CFA6:75583707-76038617	+	+	+		
CFA10:18252019-18487716	+	+	+		
CFA12:48851593-49019024	+	+	+		+
CFA13:34034756-34423363 ^1^	+	+	+	+	+
CFA13:47122582-47327423	+	+	+	+	+
CFA15:53805540-54125636	+	+	+	+	+
CFA19:41511154-44072972	+	+	+	+	+
CFA22:7086237-7370105	+	+	+	+	
CFA22:24782790-25362284 ^2^	+	+	+		
CFA31:2880206-4129393	+	+	+	+	+
CFA32:14687852-15257168	+	+	+		
CFA34:2522638-2767316	+	+	+	+	+
CFA34:15179149-15450638	+	+	+		

^1^ Deletions at CFA13:34121352-34138888 and CFA13:34202633-34373142; ^2^ Deletion at CFA22:24939999-25103044.

**Table 2:** Genomic regions of Chinese raccoon dog (*Nyctereutes procyonoides procyonoides* Gray, NPP) Bs. Region coordinates are given according to dog (canFam3) genome assembly. NPPB1—flow-sorted Bs, NPPB2 and 3—whole microdissected Bs, NPPB4 and 8—microdissected distal part of Bs, NPPB5 and 6—microdissected proximal part of Bs, NPPB7—microdissected middle part of B, BAC—BAC clone mapping data from [20]. +—regions with >5 read positions (recovered automatically). ~—regions with <5 read positions (recovered manually).

Region	NPPB1	NPPB2	NPPB3	NPPB4	NPPB5	NPPB6	NPPB7	NPPB8	BAC
CFA1:207953-434482	+								
CFA3:7233009-7672854	+	+	+	+	~	~	~	+	
CFA3:30949630-31192570	+	+	+					+	
CFA5:5213259-5517316	+					+			
CFA5:60890426-60976094	+								
CFA5:84586827-84980563	+							+	
CFA13:47079087-47478846	+							+	+
CFA14:58162796-58517967	+								
CFA15:58671318-58919401	+	+	+	~	+	+	+		
CFA16:5042848-5239664	+	~	~	~		+		+	
CFA16:50655536-50916841	+								
CFA16:51357728-51710145	+								
CFA19:28836248-29004558	+	+	+	+	~	+	+	+	
CFA19:38810088-39213802	+	+	+			+	~	+	
CFA20:24724662-25132678	+	+	+			~		+	+
CFA21:36289982-36390083	+	+	~			+	+		
CFA23:47315786-47535793	+	+	+	~	+	+	+		
CFA24:11660722-11768215	~					+			
CFA24:43488879-43732408	~	~	~			+		~	
CFA26:18769412-18964210	+								
CFA27:31691325-31793171	+					+			
CFA27:37050854-37410557	+	+	+	+	+	+	+	+	
CFA28:3855680-4240133	+	+	+			+	+		+
CFA28:7498907-7656661	+	+	+	~		+	+	+	
CFA29:28913684-29259138	+							+	
CFA32:13104843-14881357	+					+			
CFA38:1062270-1094480	+					+			

**Table 3:** B chromosome genes statistics. Genes retrieved from the Ensembl 92 database. HSA hom—number of genes with homologs in human (*Homo sapiens* L.), HSA 1-to-1—number of genes with one-to-one (i.e., single copy) orthologs in human.

Sample	Reference	Region Size, bp	Genes	HSA hom	HSA 1-to-1
VVUB	CanFam3.1	7,708,416	49	17	9
NPPB	CanFam3.1	8,510,228	100	44	36
CPYB	UMD3.1	2,355,879	9	6	4
MGOB	UMD3.1	10,456,241	107	113	81
AFLB	mm10	3,421,582	101	37	25
APEB	mm10	12,641,228	152	49	41

**Table 4:** Selected B chromosome gene functions. Neuron synapse and cell cycle proteins retrieved from DAVID Gene ontology (GO) clustering results, while differentiation and proliferation proteins are listed based on keyword match in the GO database.

	Neuron Synapse, Cell Junction	Cell Division, Microtubules	Differentiation, Proliferation
VVUB	*CTNND2*	*CENPN*	*KIT*
NPPB	*LRRC7, CXCR4, KDR, ARHGAP32*		*AICDA, APOBEC1, ARNTL, BARX2, BTBD10, COL4A3BP, CXCR4, ENPP1, GDF3, GNAS, HMGCR, JAG1, KDR, KIT, MDM4*
CPYB			*TNNI3K*
MGOB	*SDK1, SDK2, GABRA4, GABRB1, PALLD, LPP, SHANK2*	*CC2D2A, EVI5, CHFR, CCND2, TRIM67, PALLD, CDC42EP4*	*ACVR2B, BCL6, BST1, CCND2, CD38, DHCR7, DLEC1, EOMES, EVI5, FBXL5, FGFBP1, FNIP1, GABRB1, GFI1, HPSE, KIT, MYD88, PLCD1, SDK2, SERPINB9, SSBP3, SST, SSTR2, TXK, ZNF268*
AFLB	*CADPS*	*DYNC1I2, MAPRE1, MAP7, RPS6, CENPE, HAUS6, SAXO1*	*ACER2, KDM6A, MAPRE1, NCK2, RPS6*
APEB	*GRID2, UNC13A, ADGRL3*	*PIK3C3, MYO9B, HAUS8, KIF23, MVB12A*	*BST2, DDA1, GRID2, JAK3, TESPA1*

## Supplementary Materials

The following are available online at http://www.mdpi.com/2073-4425/9/8/405/s1: Supplementary Tables and Figures: Figures S1 and S2: FISH confirmation of chromosome specificity, Table S1: Sequencing statistics, Table S2: Alignment statistics, Table S3: Copy number variant calling for fox, Table S4: Regions of Siberian roe deer B chromosomes, Table S5: Regions of grey brocket deer B chromosomes; Supplementary File S2: Rainfall plots of distances between mapped read positions; Supplementary File S3: Homology data for genes found in B chromosomes of six mammal species; Supplementary File S4: Functional clustering for human one-to-one orthologs of B chromosome genes.

## References

[1] Wilson E.B. (1907). The supernumerary chromosomes of Hemiptera. Science.

[2] Liehr T., Claussen U., Starke H. (2004). Small supernumerary marker chromosomes (sSMC) in humans. Cytogenet. Genome Res..

[3] Storlazzi C.T., Lonoce A., Guastadisegni M.C., Trombetta D., D’Addabbo P., Daniele G., L’Abbate A., Macchia G., Surace C., Kok K. (2010). Gene amplification as double minutes or homogeneously staining regions in solid tumors: Origin and structure. Genome Res..

[4] Cohen S., Segal D. (2009). Extrachromosomal circular DNA in eukaryotes: Possible involvement in the plasticity of tandem repeats. Cytogenet. Genome Res..

[5] Jones R.N. (1991). B-chromosome drive. Am. Nat..

[6] Houben A. (2017). B chromosomes—A matter of chromosome drive. Front. Plant Sci..

[7] Schartl M., Nanda I., Schlupp I., Wilde B., Epplen J.T., Schmid M., Parzefall J. (1995). Incorporation of subgenomic amounts of DNA as compensation for mutational load in a gynogenetic fish. Nature.

[8] Dhar M.K., Friebe B., Koul A.K., Gill B.S. (2002). Origin of an apparent B chromosome by mutation, chromosome fragmentation and specific DNA sequence amplification. Chromosoma.

[9] Martis M.M., Klemme S., Banaei-Moghaddam A.M., Blattner F.R., Macas J., Schmutzer T., Scholz U., Gundlach H., Wicker T., Šimková H. (2012). Selfish supernumerary chromosome reveals its origin as a mosaic of host genome and organellar sequences. Proc. Natl. Acad. Sci. USA.

[10] Valente G.T., Conte M.A., Fantinatti B.E.A., Cabral-de-Mello D.C., Carvalho R.F., Vicari M.R., Kocher T.D., Martins C. (2014). Origin and evolution of B chromosomes in the cichlid fish *Astatotilapia latifasciata* based on integrated genomic analyses. Mol. Biol. Evol..

[11] Lamb J.C., Riddle N.C., Cheng Y.-M., Theuri J., Birchler J.A. (2007). Localization and transcription of a retrotransposon-derived element on the maize B chromosome. Chromosome Res..

[12] Makunin A.I., Dementyeva P.V., Graphodatsky A.S., Volobouev V.T., Kukekova A.V., Trifonov V.A. (2014). Genes on B chromosomes of vertebrates. Mol. Cytogenet..

[13] Houben A., Banaei-Moghaddam A.M., Klemme S., Timmis J.N. (2014). Evolution and biology of supernumerary B chromosomes. Cell. Mol. Life Sci..

[14] Ruban A., Schmutzer T., Scholz U., Houben A. (2017). How next-generation sequencing has aided our understanding of the sequence composition and origin of B chromosomes. Genes.

[15] Yoshida K., Terai Y., Mizoiri S., Aibara M., Nishihara H., Watanabe M., Kuroiwa A., Hirai H., Hirai Y., Matsuda Y. (2011). B chromosomes have a functional effect on female sex determination in Lake Victoria cichlid fishes. PLoS Genet.

[16] Banaei-Moghaddam A.M., Meier K., Karimi-Ashtiyani R., Houben A. (2013). Formation and expression of pseudogenes on the B chromosome of rye. Plant Cell Online.

[17] Navarro-Domínguez B., Ruiz-Ruano F.J., Cabrero J., Corral J.M., López-León M.D., Sharbel T.F., Camacho J.P.M. (2017). Protein-coding genes in B chromosomes of the grasshopper *Eyprepocnemis plorans*. Sci. Rep..

[18] Graphodatsky A.S., Kukekova A.V., Yudkin D.V., Trifonov V.A., Vorobieva N.V., Beklemisheva V.R., Perelman P.L., Graphodatskaya D.A., Trut L.N., Yang F. (2005). The proto-oncogene *C-KIT* maps to canid B-chromosomes. Chromosome Res..

[19] Yudkin D.V., Trifonov V.A., Kukekova A.V., Vorobieva N.V., Rubtsova N.V., Yang F., Acland G.M., Ferguson-Smith M.A., Graphodatsky A.S. (2007). Mapping of *KIT* adjacent sequences on canid autosomes and B chromosomes. Cytogenet. Genome Res..

[20] Duke Becker S.E., Thomas R., Trifonov V.A., Wayne R.K., Graphodatsky A.S., Breen M. (2011). Anchoring the dog to its relatives reveals new evolutionary breakpoints across 11 species of the Canidae and provides new clues for the role of B chromosomes. Chromosome Res..

[21] Makunin A.I., Kichigin I.G., Larkin D.M., O’Brien P.C.M., Ferguson-Smith M.A., Yang F., Proskuryakova A.A., Vorobieva N.V., Chernyaeva E.N., O’Brien S.J. (2016). Contrasting origin of B chromosomes in two cervids (Siberian roe deer and grey brocket deer) unravelled by chromosome-specific DNA sequencing. BMC Genom..

[22] Makunin A.I., Rajičić M., Karamysheva T.V., Romanenko S.A., Druzhkova A.S., Blagojević J., Vujošević M., Rubtsov N.B., Graphodatsky A.S., Trifonov V.A. (2018). Low-pass single-chromosome sequencing of human small supernumerary marker chromosomes (sSMCs) and *Apodemus* B chromosomes. Chromosoma.

[23] Yang F., O’Brien P.C.M., Milne B.S., Graphodatsky A.S., Solanky N., Trifonov V., Rens W., Sargan D., Ferguson-Smith M.A. (1999). A complete comparative chromosome map for the dog, red fox, and human and its integration with canine genetic maps. Genomics.

[24] Nie W., Wang J., Perelman P., Graphodatsky A.S., Yang F. (2003). Comparative chromosome painting defines the karyotypic relationships among the domestic dog, Chinese raccoon dog and Japanese raccoon dog. Chromosome Res..

[25] Trifonov V.A., Perelman P.L., Kawada S.-I., Iwasa M.A., Oda S.-I., Graphodatsky A.S. (2002). Complex structure of B-chromosomes in two mammalian species: *Apodemus peninsulae* (Rodentia) and *Nyctereutes procyonoides* (Carnivora). Chromosome Res..

[26] Yang F., Trifonov V., Ng B.L., Kosyakova N., Carter N.P., Liehr T. (2017). Generation of paint probes from flow-sorted and microdissected chromosomes. Fluorescence in Situ Hybridization (FISH) Application Guide.

[27] Telenius H., Carter N.P., Bebb C.E., Nordenskjo¨ld M., Ponder B.A.J., Tunnacliffe A. (1992). Degenerate oligonucleotide-primed PCR: General amplification of target DNA by a single degenerate primer. Genomics.

[28] Martin M. (2011). Cutadapt removes adapter sequences from high-throughput sequencing reads. EMBnet. J..

[29] Li H. (2013). Aligning sequence reads, clone sequences and assembly contigs with BWA-MEM. arXiv.

[30] Kukekova A., Johnson J., Xiang X., Feng S., Liu S., Rando H., Kharlamova A., Herbeck Y., Serdyukova N., Xiong Z. (2018). Red fox genome assembly identifies genomic regions associated with tame and aggressive behaviors. Nat. Ecol. Evol..

[31] Abyzov A., Urban A.E., Snyder M., Gerstein M. (2011). CNVnator: An approach to discover, genotype, and characterize typical and atypical CNVs from family and population genome sequencing. Genome Res..

[32] Zerbino D.R., Achuthan P., Akanni W., Amode M.R., Barrell D., Bhai J., Billis K., Cummins C., Gall A., Girón C.G. (2017). Ensembl 2018. Nucleic Acids Res..

[33] Durinck S., Spellman P.T., Birney E., Huber W. (2009). Mapping identifiers for the integration of genomic datasets with the R/Bioconductor package biomaRt. Nat. Protoc..

[34] Huang D.W., Sherman B.T., Lempicki R.A. (2009). Bioinformatics enrichment tools: Paths toward the comprehensive functional analysis of large gene lists. Nucleic Acids Res..

[35] Huang D.W., Sherman B.T., Lempicki R.A. (2009). Systematic and integrative analysis of large gene lists using DAVID bioinformatics resources. Nat. Protoc..

[36] Li Y., Lee C., Chang W., Li S.-Y., Lin C. (2002). Isolation and identification of a novel satellite DNA family highly conserved in several Cervidae species. Chromosoma.

[37] Trifonov V.A., Dementyeva P.V., Larkin D.M., O’Brien P.C., Perelman P.L., Yang F., Ferguson-Smith M.A., Graphodatsky A.S. (2013). Transcription of a protein-coding gene on B chromosomes of the Siberian roe deer (*Capreolus pygargus*). BMC Biol..

[38] Wang Y., Wang S.-Q., Wang L.-P., Yao Y.-H., Ma C.-Y., Ding J.-F., Ye J., Meng X.-M., Li J.-J., Xu R.-X. (2017). Overexpression of cardiac-specific kinase TNNI3K promotes mouse embryonic stem cells differentiation into cardiomyocytes. Cell. Physiol. Biochem..

[39] Lamatsch D.K., Trifonov V., Schories S., Epplen J.T., Schmid M., Schartl M. (2011). Isolation of a cancer-associated microchromosome in the sperm-dependent parthenogen *Poecilia formosa*. Cytogenet. Genome Res..

[40] Pieńkowska-Schelling A., Schelling C., Zawada M., Yang F., Bugno M., Ferguson-Smith M. (2008). Cytogenetic studies and karyotype nomenclature of three wild canid species: Maned wolf (*Chrysocyon brachyurus*), bat-eared fox (*Otocyon megalotis*) and fennec fox (*Fennecus zerda*). Cytogenet. Genome Res..

[41] Hsu T.C., Benirschke K. (1973). An Atlas of Mammalian Chromosomes.

[42] Bhatnagar V.S. (1973). Microchromosomes in the somatic cells of *Vulpes bengalensis* Shaw. Chromosome Inf. Serv..

[43] Chiarelli A.B. (1975). The chromosomes of the Canidae. The Wild Canids, Their Systematics, Behavioral Ecology, and Evolution.

[44] Gustavsson I., Sundt C.O. (1965). Chromosome complex of the family Canidae. Hereditas.

[45] Moore W., Elder R.L. (1965). Chromosomes of the fox. J. Hered..

[46] Trut L.N. (1999). Early Canid Domestication: The Farm-Fox Experiment: Foxes bred for tamability in a 40-year experiment exhibit remarkable transformations that suggest an interplay between behavioral genetics and development. Am. Sci..

[47] Volobujev V.T., Radzhabli S.I., Belyaeva E.S. (1976). Investigation of the nature and the role of additional chromosomes in silver foxes. III. Replication pattern in additional chromosomes. Genetika.

[48] Radzhabli S.I., Isaenko A.A., Volobujev V.T. (1978). Investigation of the nature and the role of additional chromosomes in silver fox. IV. B-chromosomes behaviour in meiosis. Genetika.

[49] Świtoński M., Gustavsson I., Höjer K., Plöen L. (1987). Synaptonemal complex analysis of the B-chromosomes in spermatocytes of the silver fox (*Vulpes fulvus* Desm.). Cytogenet. Genome Res..

[50] Basheva E.A., Torgasheva A.A., Sakaeva G.R., Bidau C., Borodin P.M. (2010). A-and B-chromosome pairing and recombination in male meiosis of the silver fox (*Vulpes vulpes* L., 1758, Carnivora, Canidae). Chromosome Res..

[51] Bugno-Poniewierska M., Solek P., Wronski M., Potocki L., Jezewska-Witkowska G., Wnuk M. (2014). Genome organization and DNA methylation patterns of B chromosomes in the red fox and Chinese raccoon dogs. Hereditas.

[52] Kociucka B., Sosnowski J., Kubiak A., Nowak A., Pawlak P., Szczerbal I. (2013). Three-dimensional positioning of B chromosomes in fibroblast nuclei of the red fox and the Chinese raccoon dog. Cytogenet. Genome Res..

[53] Mäkinen A., Fredga K. Banding analyses of the somatic chromosomes of raccoon dogs, *Nyctereutes procyonoides*, from Finland. Proceedings of the 4th European Colloquium on Cytogenetics of Domestic Animals.

[54] Yosida T.H., Wada M.Y., Ward O.G. (1983). Karyotype of a Japanese raccoon dog with 40 chromosomes including two supernumeraries. Proc. Jpn. Acad. Ser. B.

[55] Wada M.Y., Lim Y., Wurster-Hill D.H. (1991). Banded karyotype of a wild-caught male Korean raccoon dog, *Nyctereutes procyonoides koreensis*. Genome.

[56] Ward O.G., Wurster-Hill D.H., Ratty F.J., Song Y. (1987). Comparative cytogenetics of Chinese and Japanese raccoon dogs, *Nyctereutes procyonoides*. Cytogenet. Genome Res..

[57] Yosida T.H., Wada M.Y. (1984). Cytogenetical studies on the Japanese raccoon dog. VI. Distribution of B-chromosomes in 1372 cells from 13 specimens, with special note on the frequency of the Robertsonian fission. Proc. Jpn. Acad. Ser. B Phys. Biol. Sci..

[58] Yosida T.H., Wada M.Y. (1985). Cytogenetical studies on the Japanese raccoon dog. VIII. B-chromosomes observed in the spermatogonial metaphase cells. Proc. Jpn. Acad. Ser. B.

[59] Szczerbal I., Switonski M. (2003). B chromosomes of the Chinese raccoon dog (*Nyctereutes procyonoides procyonoides* Gray) contain inactive NOR-like sequences. Caryologia.

[60] Wurster-Hill D.H., Ward O.G., Kada H., Whittemore S. (1986). Banded chromosome studies and B chromosomes in wild-caught raccoon dogs, *Nyctereutes procyonoides viverrinus*. Cytogenet. Genome Res..

[61] Shi L., Tang L., Ma K., Ma C. (1988). Synaptonemal complex formation among supernumerary B chromosomes: An electron microscopic study on spermatocytes of Chinese raccoon dogs. Chromosoma.

[62] Wurster-Hill D.H., Ward O.G., Davis B.H., Park J.P., Moyzis R.K., Meyne J. (1988). Fragile sites, telomeric DNA sequences, B chromosomes, and DNA content in raccoon dogs, *Nyctereutes procyonoides*, with comparative notes on foxes, coyote, wolf, and raccoon. Cytogenet. Genome Res..

[63] Li Y.M., Zhang Y., Zhu W.J., Yan S.Q., Sun J.H. (2016). Identification of polymorphisms and transcriptional activity of the proto-oncogene *KIT* located on both autosomal and B chromosomes of the Chinese raccoon dog. Genet. Mol. Res..

[64] Ma W., Gabriel T.S., Martis M.M., Gursinsky T., Schubert V., Vrána J., Doležel J., Grundlach H., Altschmied L., Scholz U. (2017). Rye B chromosomes encode a functional Argonaute-like protein with in vitro slicer activities similar to its A chromosome paralog. New Phytol..

[65] Sugimoto Y., Morita R., Amano K., Shah P.U., Pascual-Castroviejo I., Khan S., Delgado-Escueta A.V., Yamakawa K. (2001). T-STAR gene: Fine mapping in the candidate region for childhood absence epilepsy on 8q24 and mutational analysis in patients. Epilepsy Res..

[66] Posey A.D., Demonbreun A., McNally E.M. (2011). Ferlin proteins in myoblast fusion and muscle growth. Curr. Top. Dev. Biol..

[67] Turtoi A., Blomme A., Bellahcène A., Gilles C., Hennequière V., Peixoto P., Bianchi E., Noel A., De Pauw E., Lifrange E. (2013). Myoferlin is a key regulator of EGFR activity in breast cancer. Cancer Res..

