# Supplementary material for: Sequencing of Supernumerary Chromosomes of Red Fox and Raccoon Dog Confirms a Non-Random Gene Acquisition by B Chromosomes
Source: Genes (Basel). 2018 Aug 10;9(8):405. doi: 10.3390/genes9080405 (PMC6116037; doi:10.3390/genes9080405)
Supplement: Supplementary file 1 [file genes-09-00405-s001.zip › S1 Tables and Figures v5.docx]

Supplementary Tables and Figures

**Table S1.** Sequencing statistics. NPP - Chinese raccoon dog (*Nyctereutes procyonoides procyonoides*), VVU - red fox (*Vulpes vulpes*)

| **sample** | **SRA accession** | **Isolation** | **Amplifi-**  **cation** | **Read length** | **Total reads** | **Trimmed reads** | **Total bp** | **Trimmed bp** |
| --- | --- | --- | --- | --- | --- | --- | --- | --- |
| **NPP6** | SAMN09488272 | sorting | DOP | 151 | 299338 | 295804 | 36119256 | 34052507 |
| **NPPB1** | SAMN09488273 | sorting | DOP | 151 | 736856 | 722794 | 78135334 | 73567431 |
| **NPPB2** | SAMN09488274 | dissection | WGA | 301 | 886846 | 513102 | 237889389 | 125488476 |
| **NPPB3** | SAMN09488275 | dissection | WGA | 301 | 905484 | 771036 | 235315361 | 194540481 |
| **NPPB4_dist** | SAMN09488276 | dissection | WGA | 301 | 875166 | 606696 | 225261639 | 144121525 |
| **NPPB5_prox** | SAMN09488277 | dissection | WGA | 301 | 1405480 | 820248 | 365263620 | 188759555 |
| **NPPB6_prox** | SAMN09488278 | dissection | WGA | 251 | 1953304 | 1935846 | 437803098 | 432474962 |
| **NPPB7_mid** | SAMN09488279 | dissection | WGA | 251 | 1266562 | 1255630 | 289570202 | 286363747 |
| **NPPB8_dist** | SAMN09488280 | dissection | WGA | 251 | 399220 | 394364 | 91054239 | 89698484 |
| **VVU3** | SAMN09488281 | dissection | WGA | 151 | 166540 | 150378 | 22935480 | 17495683 |
| **VVUB2** | SAMN09488282 | sorting | DOP | 161 | 753080 | 718720 | 121245880 | 95854456 |
| **VVUB3** | SAMN09488283 | dissection | WGA | 151 | 155552 | 145356 | 21440128 | 16889797 |
| **VVUB5** | SAMN09488284 | dissection | WGA | 151 | 163518 | 154678 | 22666966 | 18429946 |
| **VVUB6** | SAMN09488285 | dissection | WGA | 151 | 138566 | 123394 | 18551602 | 13714757 |

**Table S2.** Alignment statistics

| **sample** | **Reads aligned** | **Reads contaminant** | **Reads retained** | **Read positions** | **Position total bp** | **Position mean bp** | **Reads per position** |
| --- | --- | --- | --- | --- | --- | --- | --- |
| **NPP6** | 302539 | 102807 | 148901 | 40365 | 6156614 | 152.52 | 3.69 |
| **NPPB1** | 739974 | 109242 | 552218 | 94354 | 13504484 | 143.13 | 5.85 |
| **NPPB2** | 514197 | 148404 | 49274 | 4277 | 151892 | 35.51 | 11.52 |
| **NPPB3** | 772682 | 203050 | 81393 | 4276 | 144832 | 33.87 | 19.03 |
| **NPPB4_dist** | 632738 | 423133 | 54499 | 1918 | 79631 | 41.52 | 28.41 |
| **NPPB5_prox** | 842768 | 558990 | 64535 | 1960 | 72600 | 37.04 | 32.93 |
| **NPPB6_prox** | 1984478 | 984899 | 282500 | 8570 | 519552 | 60.62 | 32.96 |
| **NPPB7_mid** | 1299804 | 749190 | 139365 | 3348 | 189700 | 56.66 | 41.63 |
| **NPPB8_dist** | 408487 | 235872 | 58864 | 2152 | 197441 | 91.75 | 27.35 |
| **NPV3** | 339937 | 21077 | 26837 | 2538 | 243078 | 95.78 | 10.57 |
| **VVU3** | 152380 | 15771 | 13438 | 5962 | 693314 | 116.29 | 2.25 |
| **VVUB2** | 746259 | 135589 | 481448 | 16424 | 2402473 | 146.28 | 29.31 |
| **VVUB3** | 146933 | 30333 | 5120 | 1849 | 219912 | 118.94 | 2.77 |
| **VVUB5** | 156786 | 60196 | 3316 | 1285 | 132944 | 103.46 | 2.58 |
| **VVUB6** | 124793 | 9447 | 2177 | 741 | 81473 | 109.95 | 2.94 |

**Table S3.** Copy number variant calling based on fox whole-genome sequencing data for B-specific regions defined by isolated chromosome sequencing. norm_RD – normalized read depth estimated by CNVnator, #B – rounded number of additional copies

| **chr** | | **start** | **end** | **type** | **norm_RD** | **#B** | **p-val1** | **p-val2** | **p-val3** | **p-val4** | **q0** |
| --- | --- | --- | --- | --- | --- | --- | --- | --- | --- | --- | --- |
| 10 | 18259001 | | 18496000 | dup | 1.58092 | 1 | 0.00E+00 | 1.57E+08 | 0.00E+00 | 1.61E+08 | 0.010718 |
| 12 | 48851001 | | 49021000 | dup | 2.92929 | 4 | 0.00E+00 | 2.87E+09 | 0.00E+00 | 2.87E+09 | 0.017403 |
| 13 | 34034001 | | 34423000 | dup | 6.32089 | 11 | 0.00E+00 | 1.02E+07 | 0.00E+00 | 1.05E+07 | 0.008076 |
| 13 | 47122001 | | 47320000 | dup | 3.97359 | 6 | 0.00E+00 | 0.00E+00 | 0.00E+00 | 0.00E+00 | 0.005281 |
| 15 | 53805001 | | 54126000 | dup | 4.19087 | 6 | 0.00E+00 | 2.14E-46 | 0.00E+00 | 4.71E-46 | 0.009673 |
| 19 | 41543001 | | 42149000 | dup | 1.61801 | 1 | 0.00E+00 | 2.87E+09 | 0.00E+00 | 2.87E+09 | 0.029872 |
| 19 | 42151001 | | 42343000 | dup | 2.66788 | 3 | 0.00E+00 | 0.00E+00 | 0.00E+00 | 0.00E+00 | 0.00908 |
| 19 | 42507001 | | 42979000 | dup | 2.52267 | 3 | 0.00E+00 | 2.87E+09 | 0.00E+00 | 2.87E+09 | 0.009686 |
| 19 | 42979001 | | 42981000 | del | 0.304066 | 0 | 1.72E+04 | 3.55E-02 | 1.00E+00 | 1.00E+00 | 0.050848 |
| 19 | 42993001 | | 44073000 | dup | 2.17161 | 2 | 0.00E+00 | 2.87E+09 | 0.00E+00 | 2.87E+09 | 0.014451 |
| 22 | 7096001 | | 7371000 | dup | 2.33848 | 3 | 0.00E+00 | 2.87E+09 | 0.00E+00 | 2.87E+09 | 0.008816 |
| 22 | 24782001 | | 24865000 | dup | 2.26685 | 3 | 0.00E+00 | 2.87E+09 | 0.00E+00 | 2.87E+09 | 0.028955 |
| 22 | 25130001 | | 25369000 | dup | 1.94431 | 2 | 0.00E+00 | 2.87E+09 | 0.00E+00 | 2.87E+09 | 0.01008 |
| 31 | 2880001 | | 2933000 | dup | 9.17981 | 16 | 0.00E+00 | 2.09E-11 | 0.00E+00 | 1.20E-10 | 0.007304 |
| 31 | 2935001 | | 3014000 | dup | 10.0723 | 18 | 0.00E+00 | 2.87E+09 | 0.00E+00 | 2.87E+09 | 0.01021 |
| 31 | 3063001 | | 3258000 | dup | 7.92126 | 14 | 0.00E+00 | 5.11E-83 | 0.00E+00 | 4.46E-82 | 0.010975 |
| 31 | 3319001 | | 3360000 | del | 0.579309 | 0 | 1.57E-02 | 2.87E+09 | 3.69E-02 | 2.87E+09 | 0.288096 |
| 31 | 3700001 | | 3731000 | dup | 2.63686 | 3 | 0.00E+00 | 4.80E-72 | 0.00E+00 | 2.40E-118 | 0.013666 |
| 31 | 3808001 | | 3831000 | dup | 2.60578 | 3 | 1.76E-08 | 2.16E+01 | 1.34E-07 | 1.10E+02 | 0.009893 |
| 31 | 3932001 | | 3956000 | dup | 6.86283 | 12 | 0.00E+00 | 0.00E+00 | 0.00E+00 | 0.00E+00 | 0.008218 |
| 31 | 3958001 | | 3997000 | dup | 5.03845 | 8 | 0.00E+00 | 4.74E-192 | 0.00E+00 | 9.38E-182 | 0.00944 |
| 31 | 4046001 | | 4130000 | dup | 10.8556 | 20 | 0.00E+00 | 0.00E+00 | 0.00E+00 | 0.00E+00 | 0.008613 |
| 32 | 14686001 | | 14996000 | dup | 1.91911 | 2 | 0.00E+00 | 2.87E+09 | 0.00E+00 | 2.87E+09 | 0.027487 |
| 32 | 14997001 | | 15000000 | del | 0.179124 | 0 | 1.12E+04 | 4.83E-02 | 1.00E+00 | 1.00E+00 | 0.019608 |
| 32 | 15026001 | | 15068000 | dup | 1.55385 | 1 | 0.00E+00 | 8.00E-30 | 0.00E+00 | 5.48E-28 | 0.016082 |
| 32 | 15083001 | | 15087000 | del | 0.425912 | 0 | 1.31E+01 | 1.49E-06 | 1.11E+04 | 8.76E+00 | 0.364583 |
| 32 | 15094001 | | 15258000 | dup | 1.55216 | 1 | 0.00E+00 | 2.32E-03 | 0.00E+00 | 3.26E-03 | 0.01576 |
| 34 | 2522001 | | 2619000 | dup | 7.29304 | 13 | 0.00E+00 | 2.87E+09 | 0.00E+00 | 2.87E+09 | 0.007957 |
| 34 | 2619001 | | 2625000 | del | 0.117742 | 0 | 1.02E+00 | 6.24E-29 | 2.14E+02 | 2.24E-16 | 0.199161 |
| 34 | 2625001 | | 2767000 | dup | 3.37986 | 5 | 4.03E-09 | 3.32E-220 | 6.42E-09 | 5.57E-217 | 0.037692 |
| 34 | 15181001 | | 15451000 | dup | 1.60098 | 1 | 0.00E+00 | 2.57E-03 | 0.00E+00 | 3.16E-03 | 0.008161 |
| 5 | 70794001 | | 70974000 | dup | 1.9791 | 2 | 0.00E+00 | 2.87E+09 | 0.00E+00 | 2.87E+09 | 0.011939 |
| 6 | 75583001 | | 75745000 | dup | 1.58114 | 1 | 0.00E+00 | 1.63E-13 | 0.00E+00 | 3.07E-13 | 0.010306 |
| 6 | 76013001 | | 76038000 | dup | 1.54722 | 1 | 1.69E-08 | 4.80E+02 | 1.39E-07 | 1.67E+03 | 0.019063 |

**Table S4.** Regions of Siberian roe deer (*Capreolus pygargus*) B chromosomes recovered in cattle genome (UMD3.1)

| **chr** | | **start** | **end** | **size** | **Makunin et al 2016** | **Trifonov et al 2013** |
| --- | --- | --- | --- | --- | --- | --- |
| BTA3 | 70650384 | | 72471374 | 1820990 | Y | Y |
| BTA7 | 44214031 | | 44217843 | 3812 |  |  |
| BTA8 | 18490157 | | 18682341 | 192184 |  |  |
| BTA17 | 51998018 | | 52000075 | 2057 |  |  |
| BTA28 | 12825263 | | 12868034 | 42771 | Y |  |
| BTAX | 146352885 | | 146354544 | 1659 |  |  |
| BTAX | 148486206 | | 148778612 | 292406 |  |  |

**Table S5.** Regions of grey brocket deer (*Mazama gouazoubira*) B chromosomes recovered in cattle genome (UMD3.1)

| **chr** | | **start** | **end** | **size** | **Makunin et al 2016** |
| --- | --- | --- | --- | --- | --- |
| BTA1 | 79619021 | | 80370209 | 751188 | Y |
| BTA1 | 131326634 | | 131382604 | 55970 | Y |
| BTA3 | 51069589 | | 51165289 | 95700 | Y |
| BTA3 | 92286020 | | 92470023 | 184003 | Y |
| BTA5 | 106241465 | | 106297937 | 56472 | Y |
| BTA6 | 66762561 | | 68453869 | 1691308 | Y |
| BTA6 | 71708989 | | 71933160 | 224171 | Y |
| BTA6 | 99798140 | | 100020983 | 222843 | Y |
| BTA6 | 115408992 | | 115949925 | 540933 | Y |
| BTA7 | 13962313 | | 14038397 | 76084 | Y |
| BTA7 | 24046195 | | 24313132 | 266937 | Y |
| BTA7 | 89345800 | | 89390817 | 45017 |  |
| BTA8 | 12302 | | 931659 | 919357 | Y |
| BTA10 | 29003152 | | 29746114 | 742962 | Y |
| BTA11 | 107289945 | | 107305567 | 15622 |  |
| BTA14 | 7491165 | | 7950097 | 458932 | Y |
| BTA17 | 45202846 | | 45430717 | 227871 | Y |
| BTA18 | 233703 | | 321208 | 87505 |  |
| BTA19 | 58466358 | | 58791098 | 324740 | Y |
| BTA22 | 1981333 | | 2115675 | 134342 | Y |
| BTA22 | 11502841 | | 11902912 | 400071 | Y |
| BTA23 | 8649839 | | 8743717 | 93878 | Y |
| BTA23 | 50632595 | | 50655552 | 22957 | Y |
| BTA25 | 35488023 | | 35689879 | 201856 | Y |
| BTA25 | 40178112 | | 40495170 | 317058 | Y |
| BTA28 | 3170279 | | 3786379 | 616100 | Y |
| BTA28 | 13566921 | | 13618008 | 51087 | Y |
| BTA29 | 33888399 | | 34627325 | 738926 | Y |
| BTA29 | 48337280 | | 49220745 | 883465 | Y |
| BTAX | 117081515 | | 117090401 | 8886 |  |

**
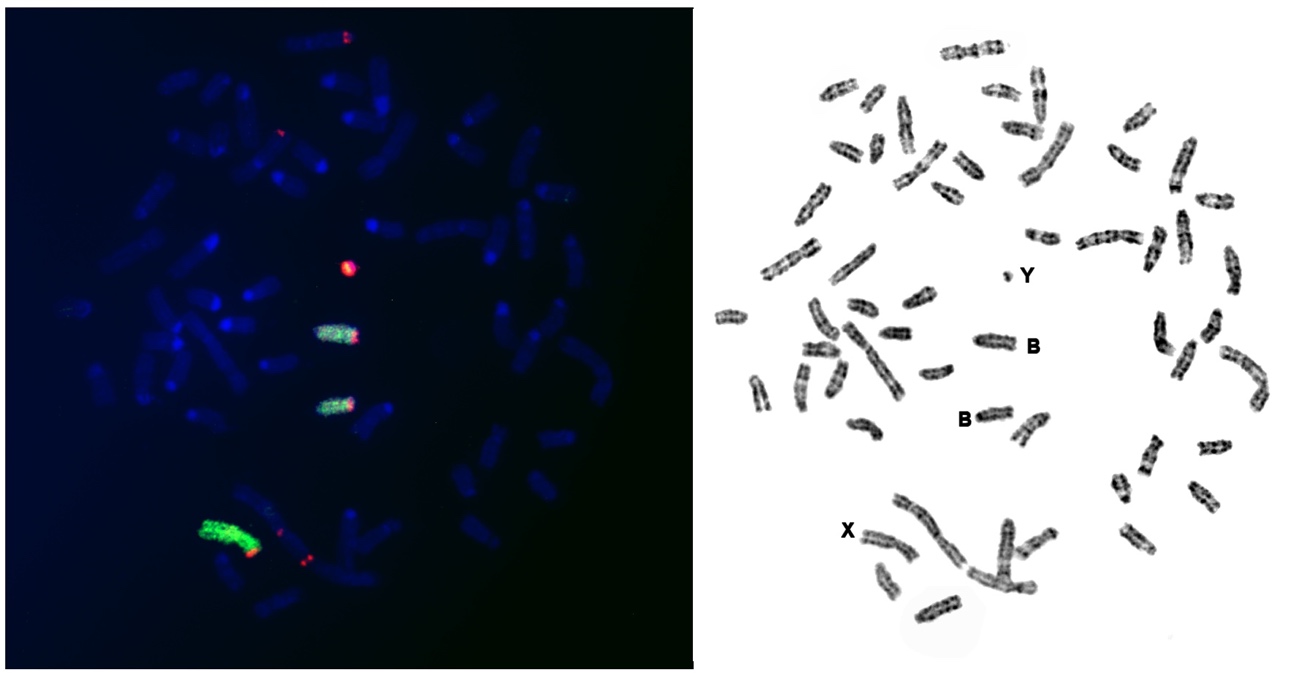
**

**Figure S1.** FISH of flow-sorted Chinese raccoon dog (*Nyctereutes procyonoides procyonoides*, NPP) B chromosome (NPPB1 in this study, green) and Y chromosome (red) probes on Chinese raccoon dog metaphase chromosomes. Staining of B chromosomes with Y chromosome probe suggests the presence of common repetitive elements.


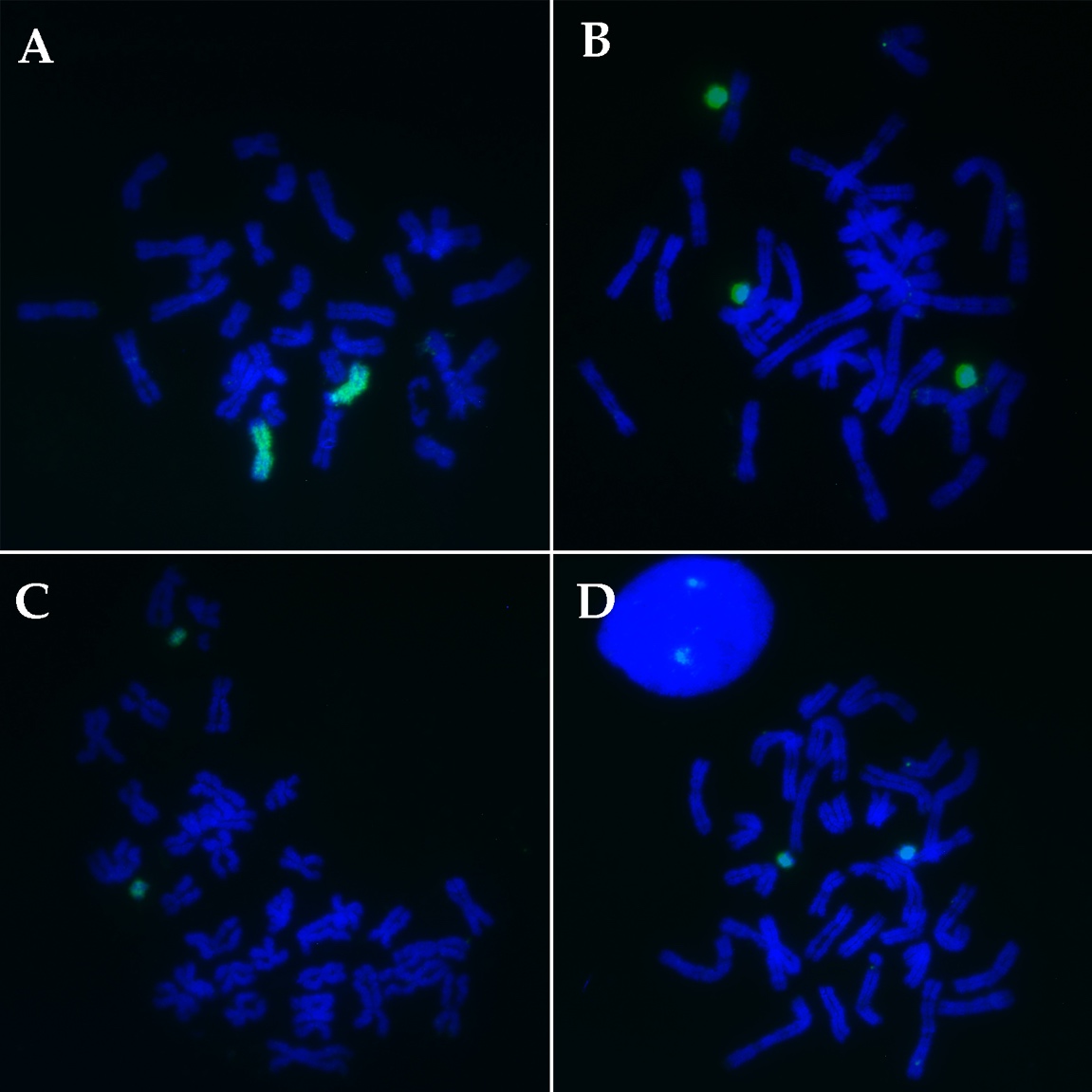


**Figure S2.** FISH of red fox (*Vulpes vulpes*, VVU) chromosome probes (green) on red fox metaphase chromosomes. Samples (A) VVU3, (B) VVUB3, (C) VVUB5, (D) VVUB6.
