# Supplementary material for: Sequencing of Supernumerary Chromosomes of Red Fox and Raccoon Dog Confirms a Non-Random Gene Acquisition by B Chromosomes
Source: Genes (Basel). 2018 Aug 10;9(8):405. doi: 10.3390/genes9080405 (PMC6116037; doi:10.3390/genes9080405)
Supplement: Supplementary file 1 [file genes-09-00405-s001.zip › S2_reg_plot.pdf]

NPP6.CanFam3

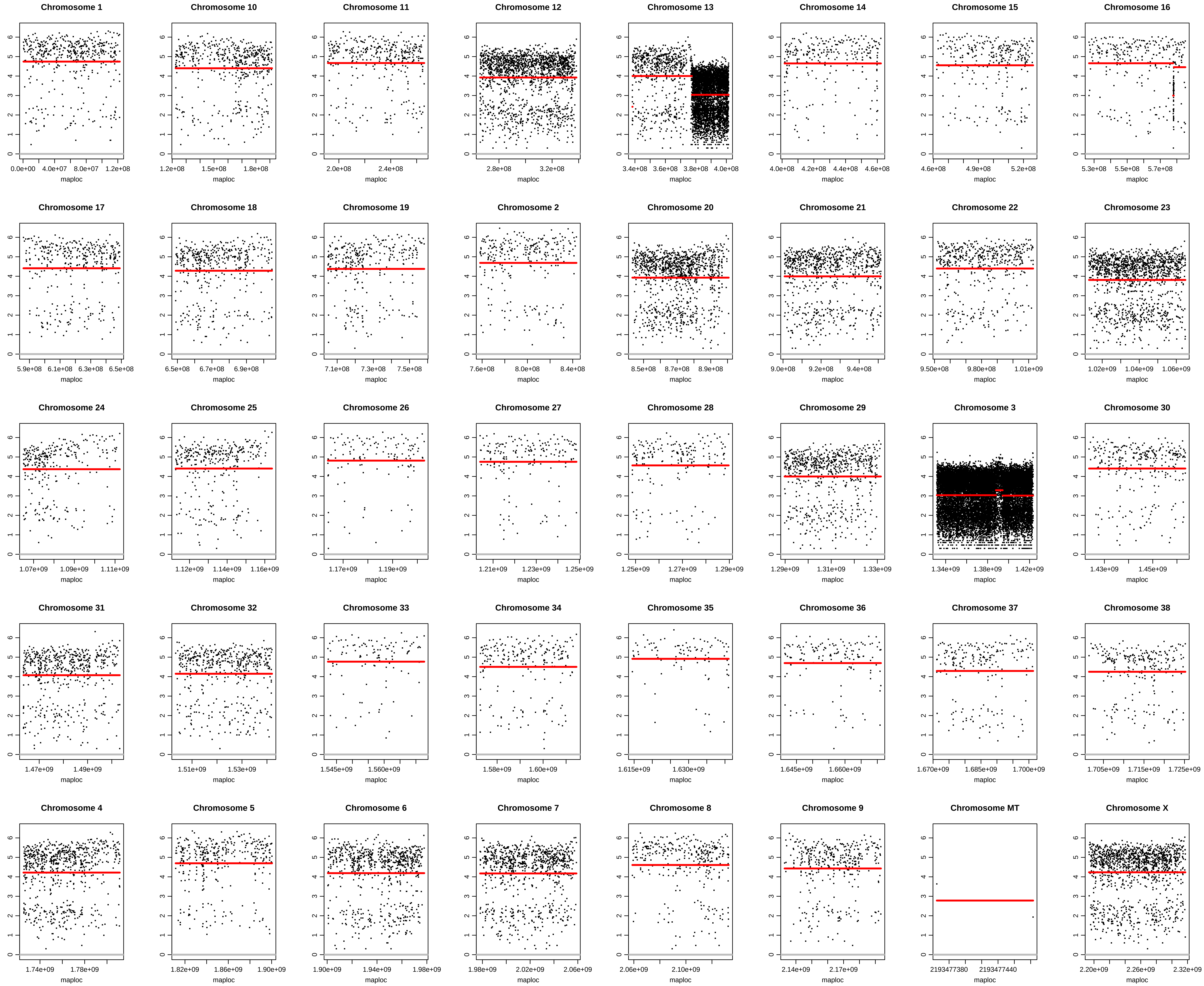

VVU3.CanFam3

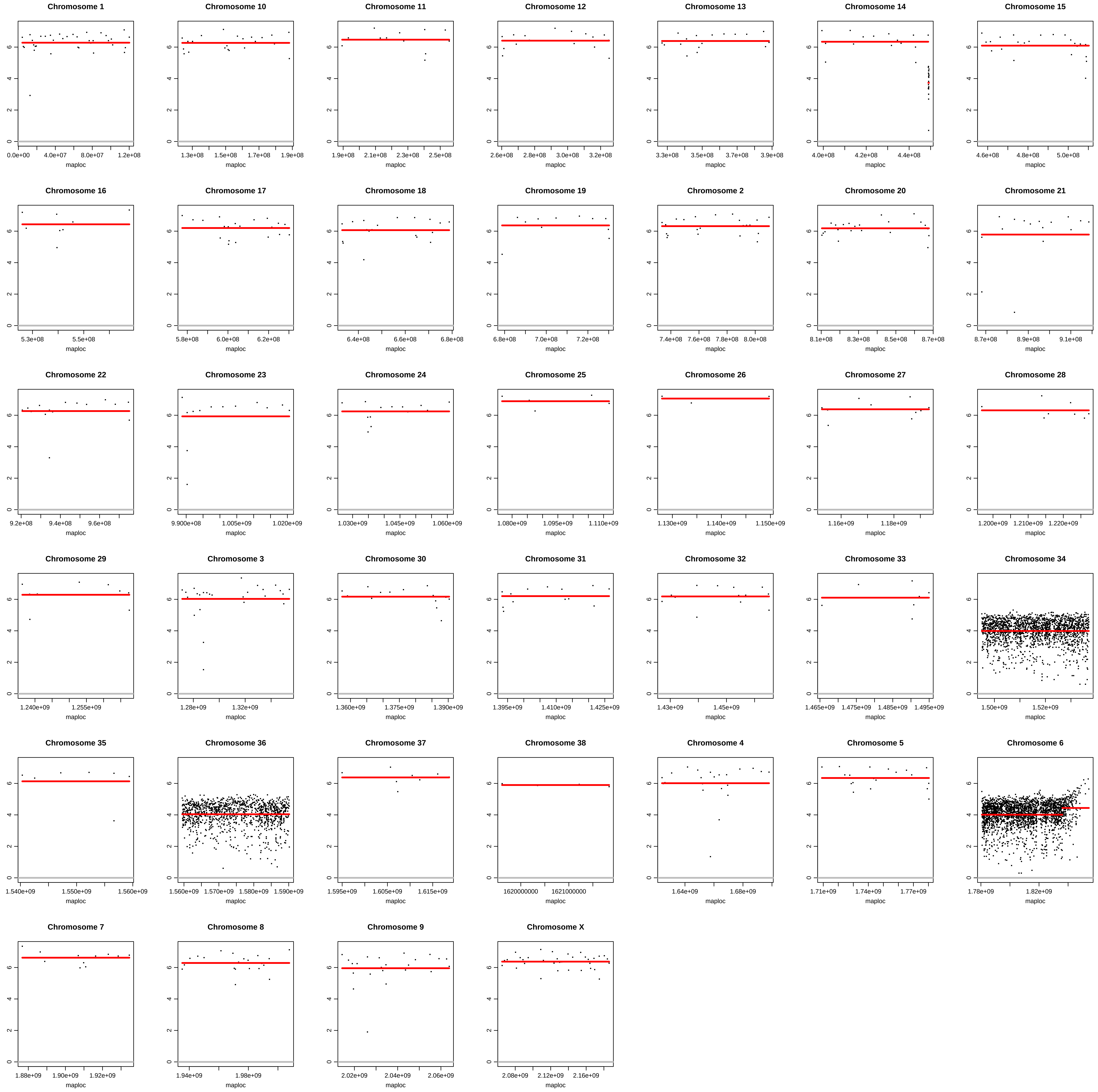

VVUB2.CanFam3

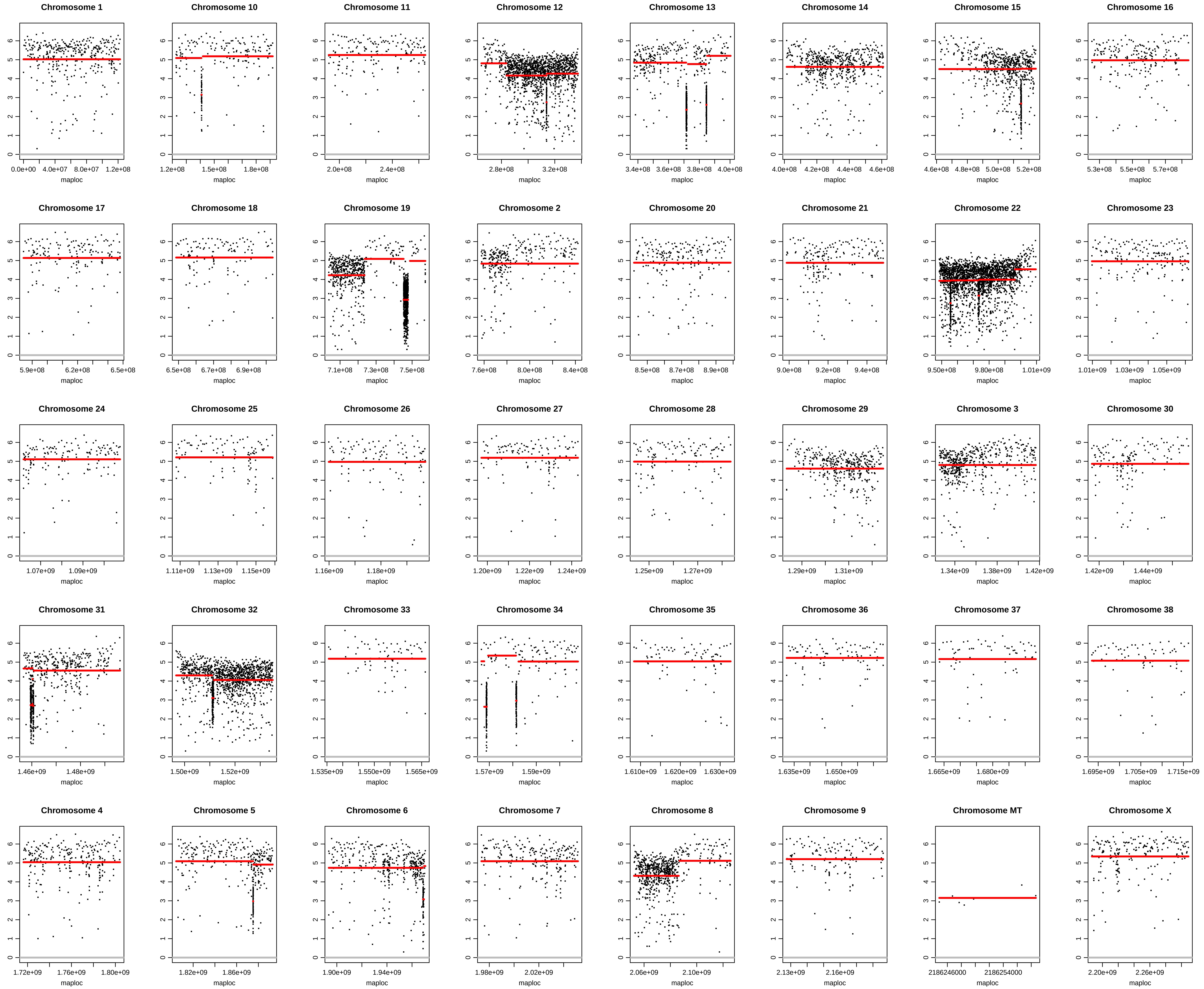

VVUB3.CanFam3

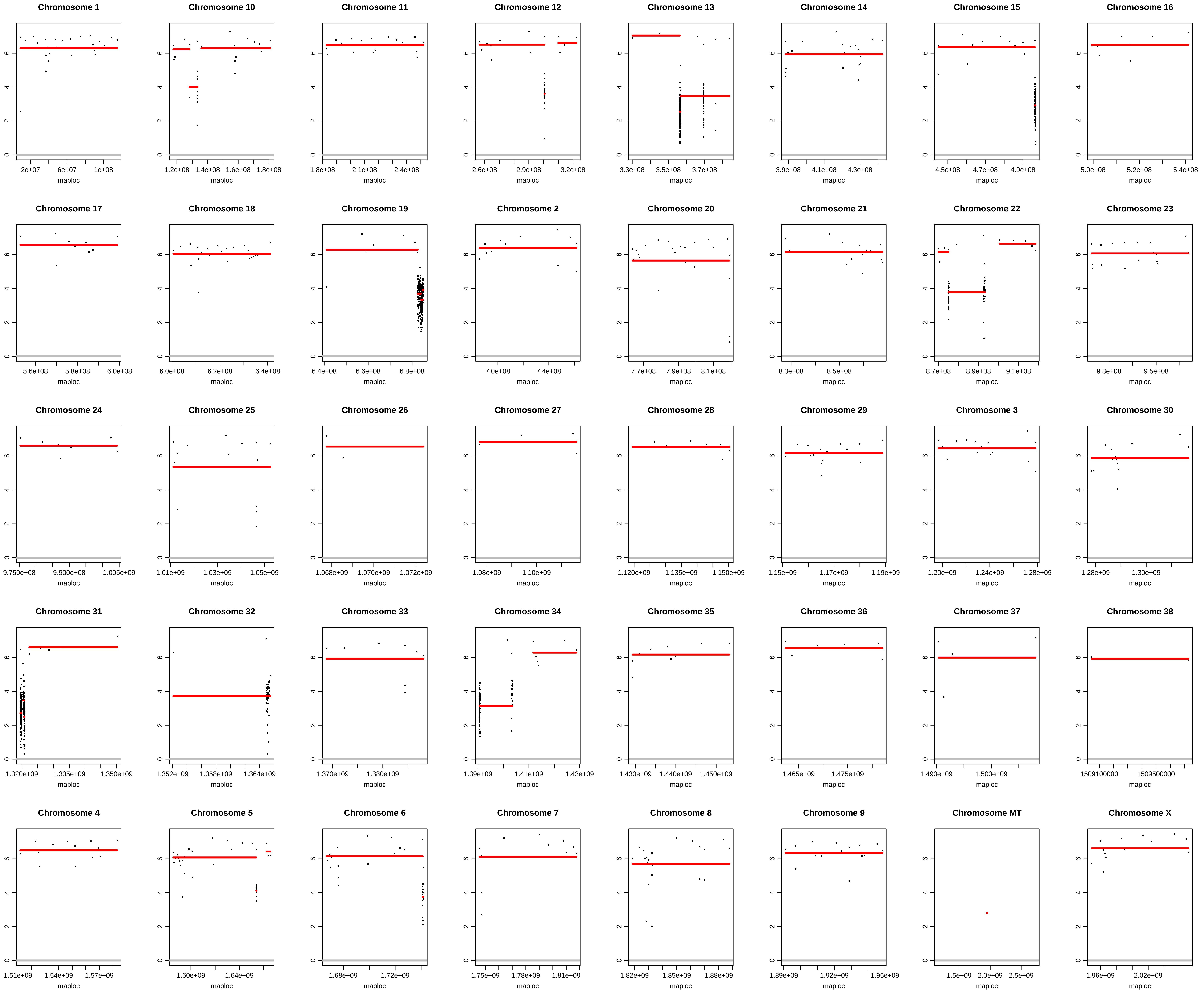

VVUB5.CanFam3

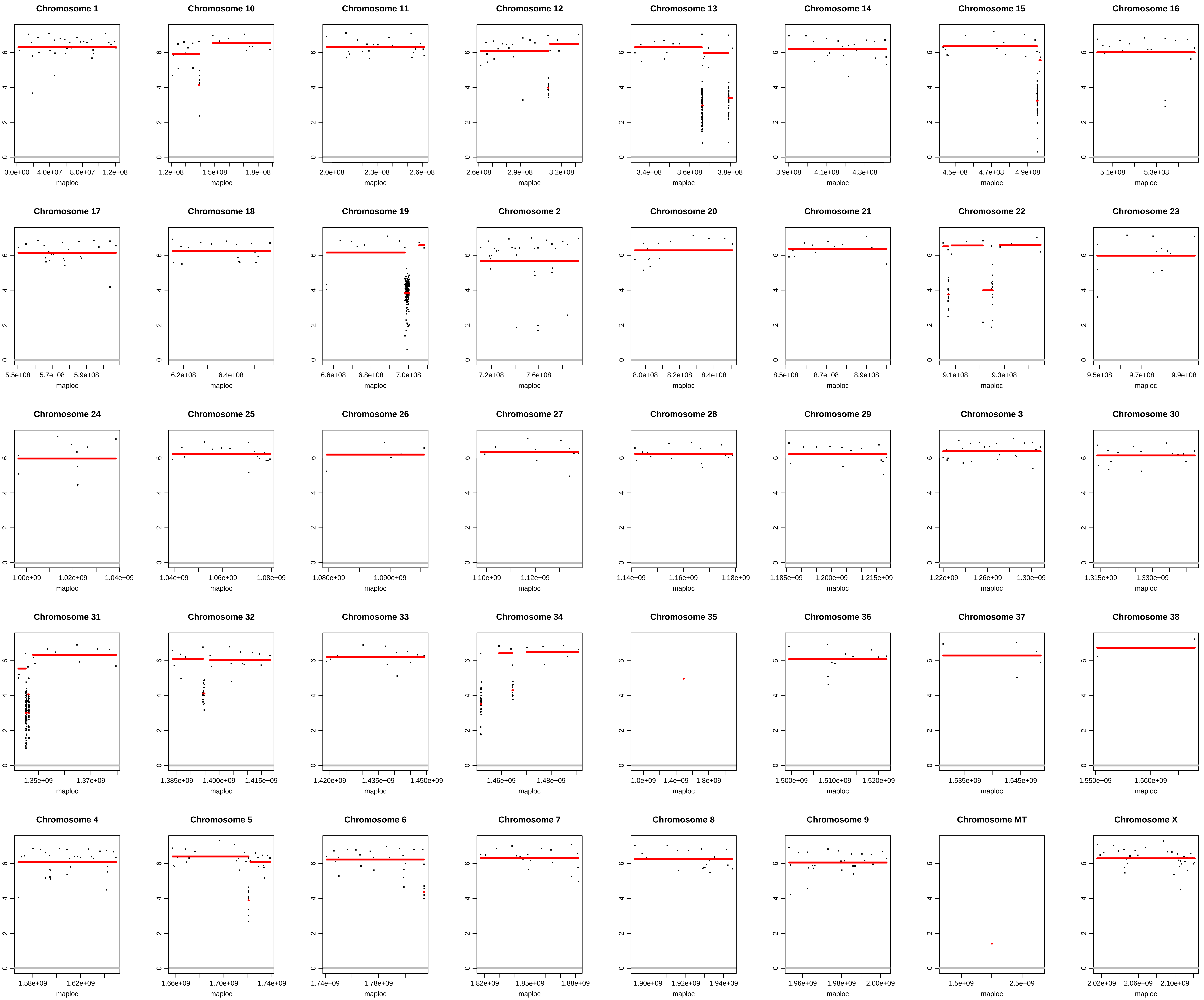

VVUB6.CanFam3

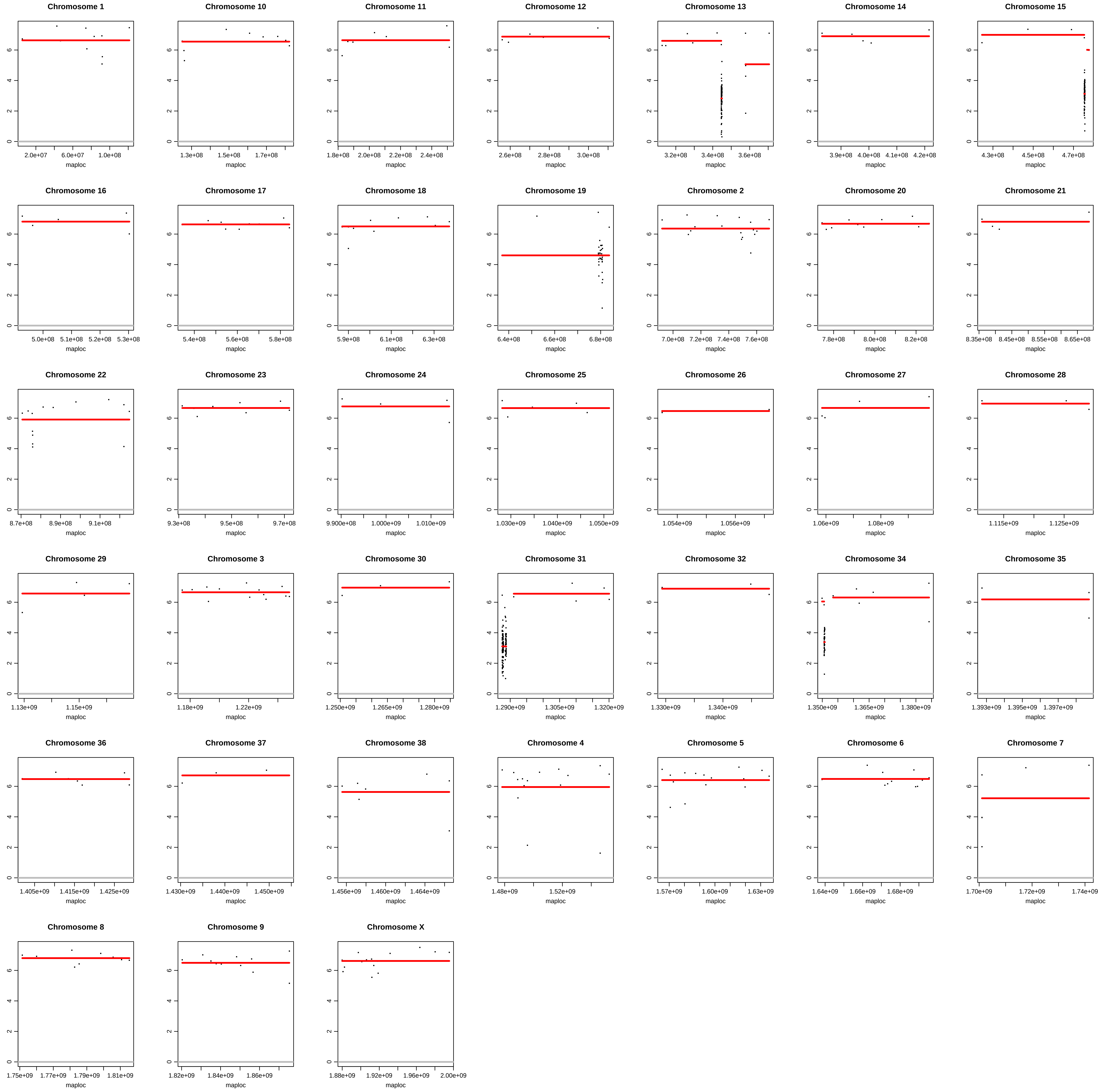

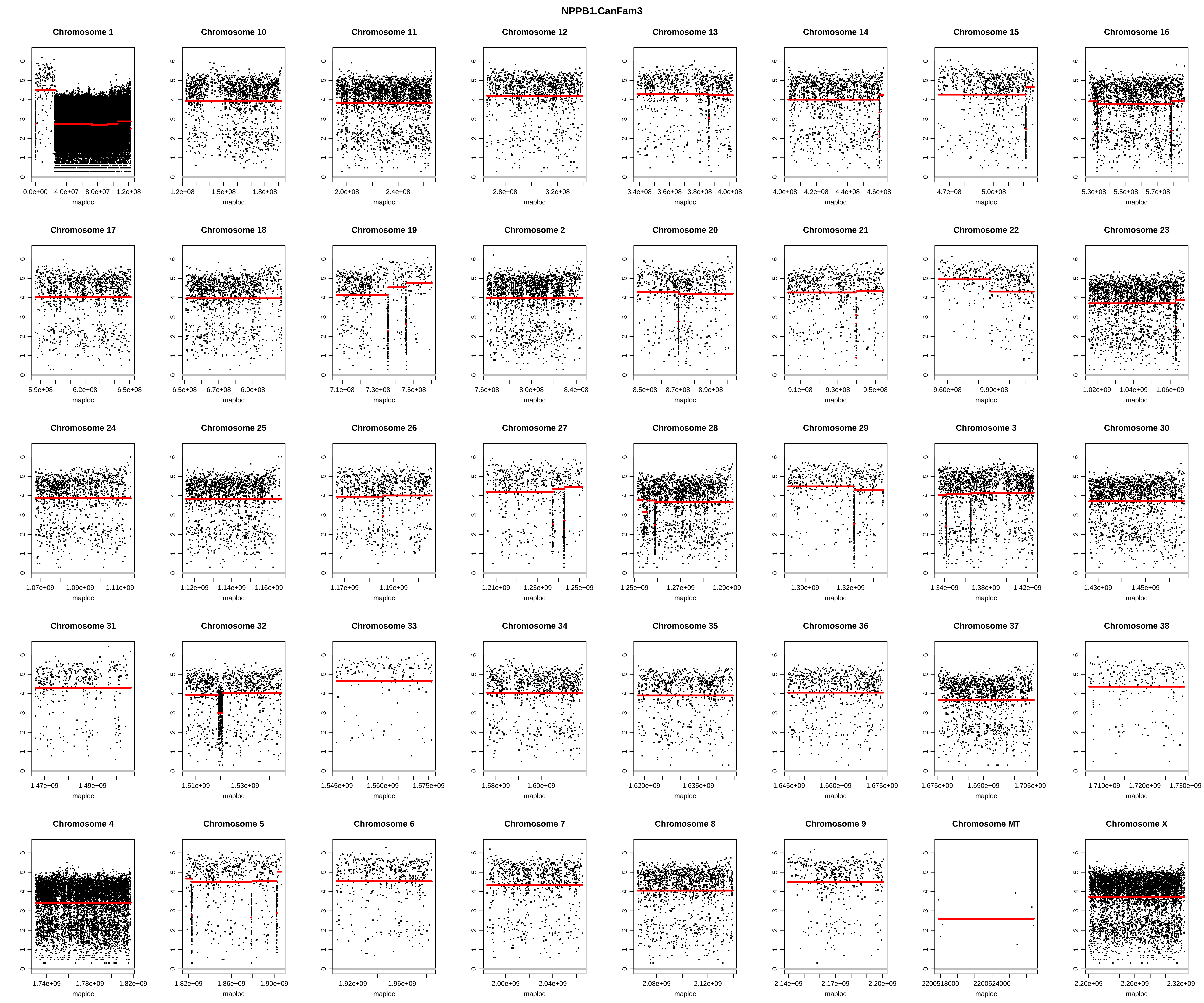
NPPB1.CanFam3
Chromosome 1
0.0e+00 4.0e+07 8.0e+07 1.2e+08
Chromosome 10
1.2e+08 1.5e+08 1.8e+08
Chromosome 11
2.0e+08 2.4e+08
Chromosome 12
2.8e+08 3.2e+08
Chromosome 13
3.4e+08 3.6e+08 3.8e+08 4.0e+08
Chromosome 14
4.0e+08 4.2e+08 4.4e+08 4.6e+08
Chromosome 15
4.7e+08 5.0e+08
Chromosome 16
5.3e+08 5.5e+08 5.7e+08
Chromosome 17
5.9e+08 6.2e+08 6.5e+08
Chromosome 18
6.5e+08 6.7e+08 6.9e+08
Chromosome 19
7.1e+08 7.3e+08 7.5e+08
Chromosome 2
7.6e+08 8.0e+08 8.4e+08
Chromosome 20
8.5e+08 8.7e+08 8.9e+08
Chromosome 21
9.1e+08 9.3e+08 9.5e+08
Chromosome 22
9.60e+08 9.90e+08
Chromosome 23
1.02e+09 1.04e+09 1.06e+09
Chromosome 24
1.07e+09 1.09e+09 1.11e+09
Chromosome 25
1.12e+09 1.14e+09 1.16e+09
Chromosome 26
1.17e+09 1.19e+09
Chromosome 27
1.21e+09 1.23e+09 1.25e+09
Chromosome 28
1.25e+09 1.27e+09 1.29e+09
Chromosome 29
1.30e+09 1.32e+09
Chromosome 3
1.34e+09 1.38e+09 1.42e+09
Chromosome 30
1.43e+09 1.45e+09
Chromosome 31
1.47e+09 1.49e+09
Chromosome 32
1.51e+09 1.53e+09
Chromosome 33
1.545e+09 1.560e+09 1.575e+09
Chromosome 34
1.58e+09 1.60e+09
Chromosome 35
1.620e+09 1.635e+09
Chromosome 36
1.645e+09 1.660e+09 1.675e+09
Chromosome 37
1.675e+09 1.690e+09 1.705e+09
Chromosome 38
1.710e+09 1.720e+09 1.730e+09
Chromosome 4
1.74e+09 1.78e+09 1.82e+09
Chromosome 5
1.82e+09 1.86e+09 1.90e+09
Chromosome 6
1.92e+09 1.96e+09
Chromosome 7
2.00e+09 2.04e+09
Chromosome 8
2.08e+09 2.12e+09
Chromosome 9
2.14e+09 2.17e+09 2.20e+09
Chromosome MT
2200518000 2200524000
Chromosome X
2.20e+09 2.26e+09 2.32e+09
maploc
0 1 2 3 4 5 6

NPPB2.CanFam3

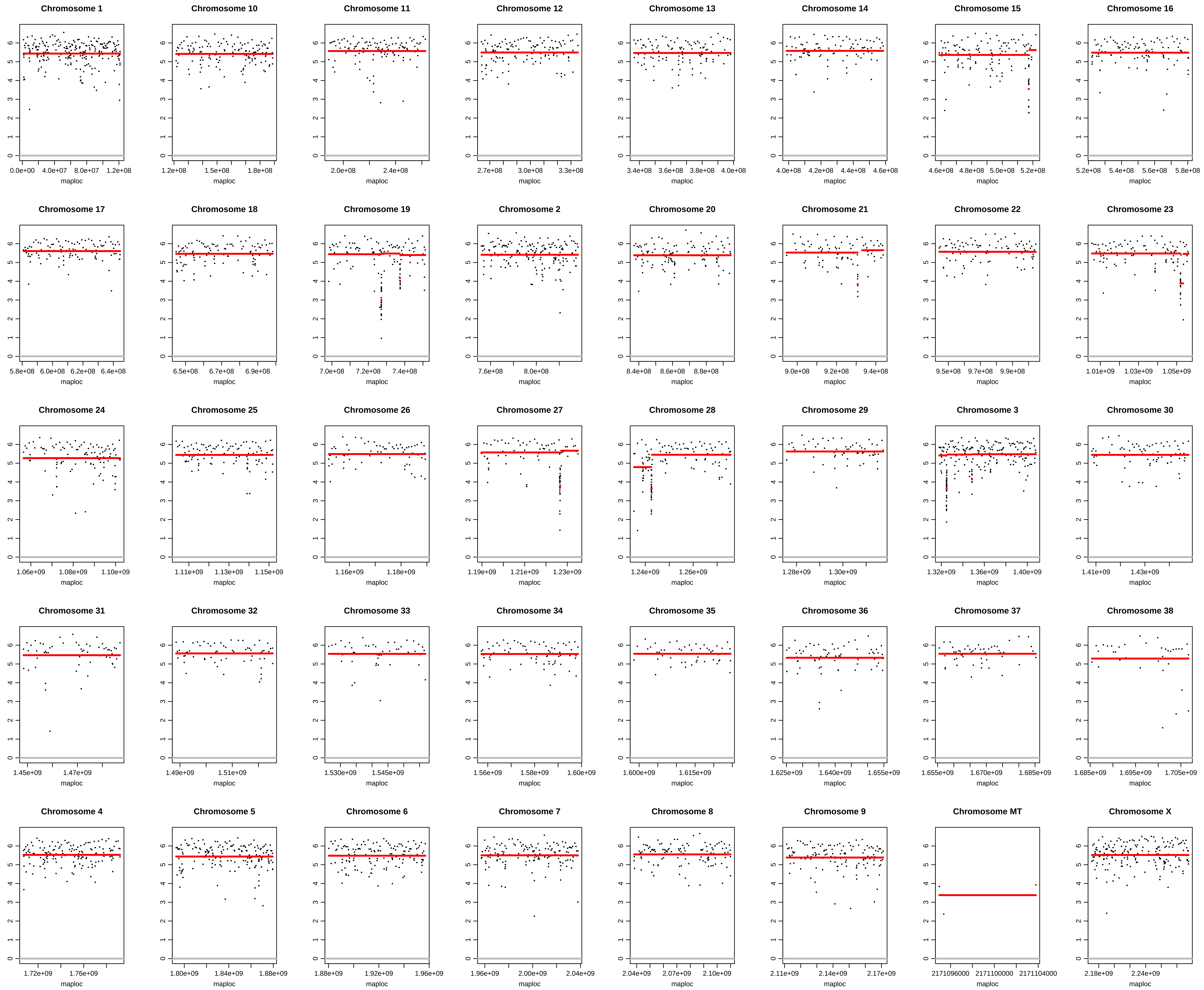

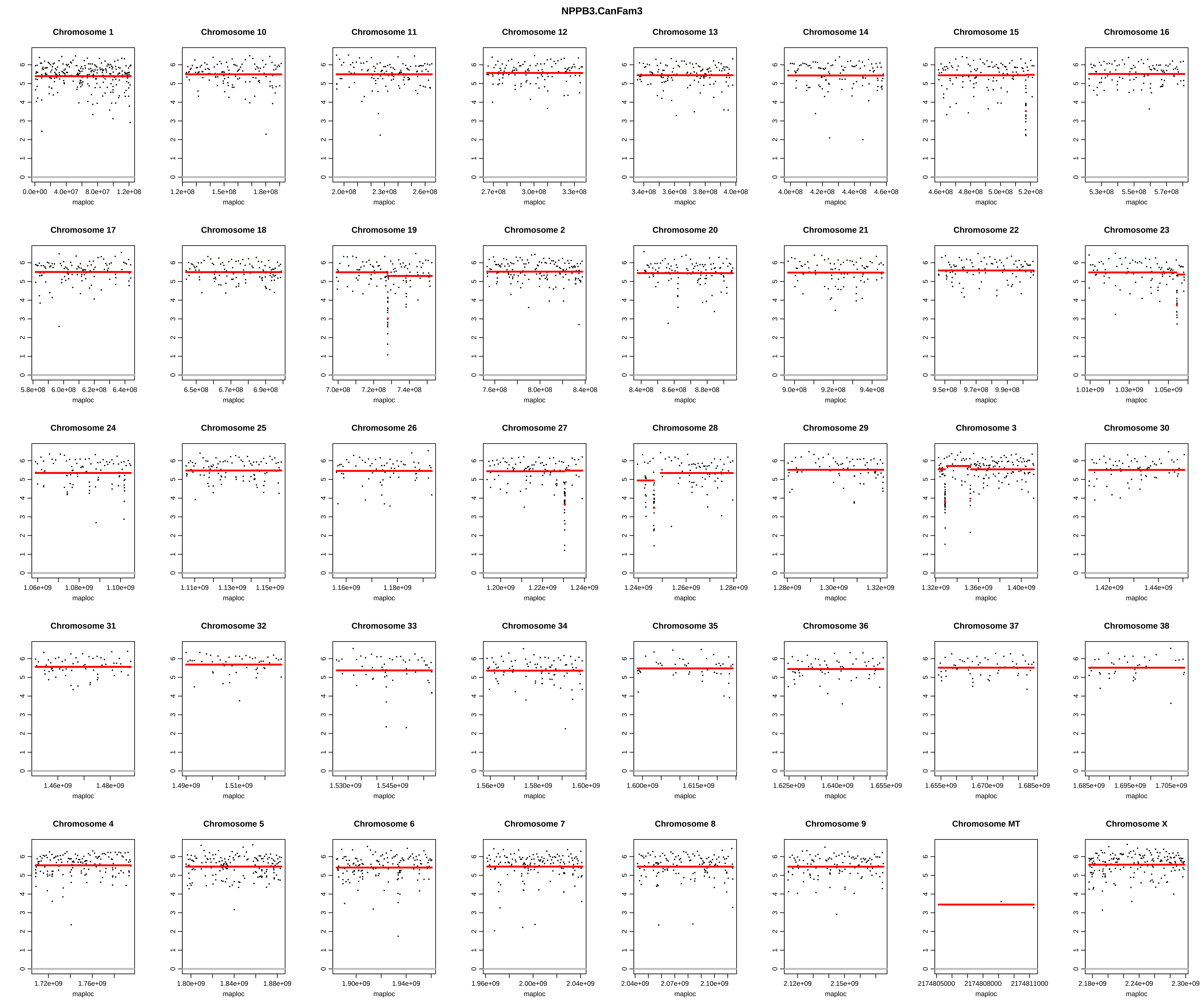
NPPB3.CanFam3
Chromosome 1
0.0e+00 4.0e+07 8.0e+07 1.2e+08
Chromosome 10
1.2e+08 1.5e+08 1.8e+08
Chromosome 11
2.0e+08 2.3e+08 2.6e+08
Chromosome 12
2.7e+08 3.0e+08 3.3e+08
Chromosome 13
3.4e+08 3.6e+08 3.8e+08 4.0e+08
Chromosome 14
4.0e+08 4.2e+08 4.4e+08 4.6e+08
Chromosome 15
4.6e+08 4.8e+08 5.0e+08 5.2e+08
Chromosome 16
5.3e+08 5.5e+08 5.7e+08
Chromosome 17
5.8e+08 6.0e+08 6.2e+08 6.4e+08
Chromosome 18
6.5e+08 6.7e+08 6.9e+08
Chromosome 19
7.0e+08 7.2e+08 7.4e+08
Chromosome 2
7.6e+08 8.0e+08 8.4e+08
Chromosome 20
8.4e+08 8.6e+08 8.8e+08
Chromosome 21
9.0e+08 9.2e+08 9.4e+08
Chromosome 22
9.5e+08 9.7e+08 9.9e+08
Chromosome 23
1.01e+09 1.03e+09 1.05e+09
Chromosome 24
1.06e+09 1.08e+09 1.10e+09
Chromosome 25
1.11e+09 1.13e+09 1.15e+09
Chromosome 26
1.16e+09 1.18e+09
Chromosome 27
1.20e+09 1.22e+09 1.24e+09
Chromosome 28
1.24e+09 1.26e+09 1.28e+09
Chromosome 29
1.28e+09 1.30e+09 1.32e+09
Chromosome 3
1.32e+09 1.36e+09 1.40e+09
Chromosome 30
1.42e+09 1.44e+09
Chromosome 31
1.46e+09 1.48e+09
Chromosome 32
1.49e+09 1.51e+09
Chromosome 33
1.530e+09 1.545e+09
Chromosome 34
1.56e+09 1.58e+09 1.60e+09
Chromosome 35
1.600e+09 1.615e+09
Chromosome 36
1.625e+09 1.640e+09 1.655e+09
Chromosome 37
1.655e+09 1.670e+09 1.685e+09
Chromosome 38
1.685e+09 1.695e+09 1.705e+09
Chromosome 4
1.72e+09 1.76e+09
Chromosome 5
1.80e+09 1.84e+09 1.88e+09
Chromosome 6
1.90e+09 1.94e+09
Chromosome 7
1.96e+09 2.00e+09 2.04e+09
Chromosome 8
2.04e+09 2.07e+09 2.10e+09
Chromosome 9
2.12e+09 2.15e+09
Chromosome MT
2174805000 2174808000 2174811000
Chromosome X
2.18e+09 2.24e+09 2.30e+09
maploc
0 1 2 3 4 5 6

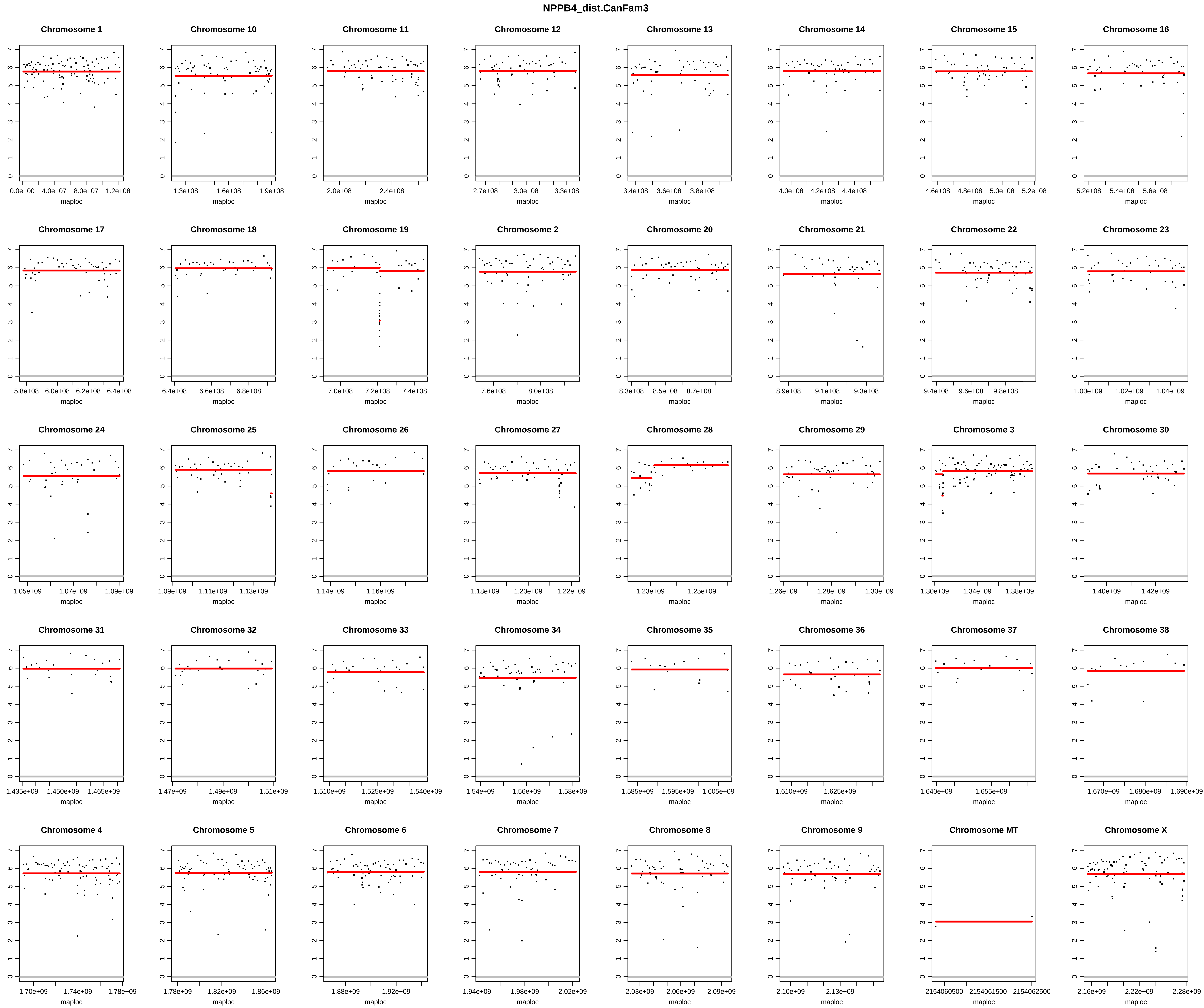
NPPB4_dist.CanFam3
Chromosome 1
Chromosome 10
Chromosome 11
Chromosome 12
Chromosome 13
Chromosome 14
Chromosome 15
Chromosome 16
Chromosome 17
Chromosome 18
Chromosome 19
Chromosome 2
Chromosome 20
Chromosome 21
Chromosome 22
Chromosome 23
Chromosome 24
Chromosome 25
Chromosome 26
Chromosome 27
Chromosome 28
Chromosome 29
Chromosome 3
Chromosome 30
Chromosome 31
Chromosome 32
Chromosome 33
Chromosome 34
Chromosome 35
Chromosome 36
Chromosome 37
Chromosome 38
Chromosome 4
Chromosome 5
Chromosome 6
Chromosome 7
Chromosome 8
Chromosome 9
Chromosome MT
Chromosome X
maploc

NPPB5_prox.CanFam3

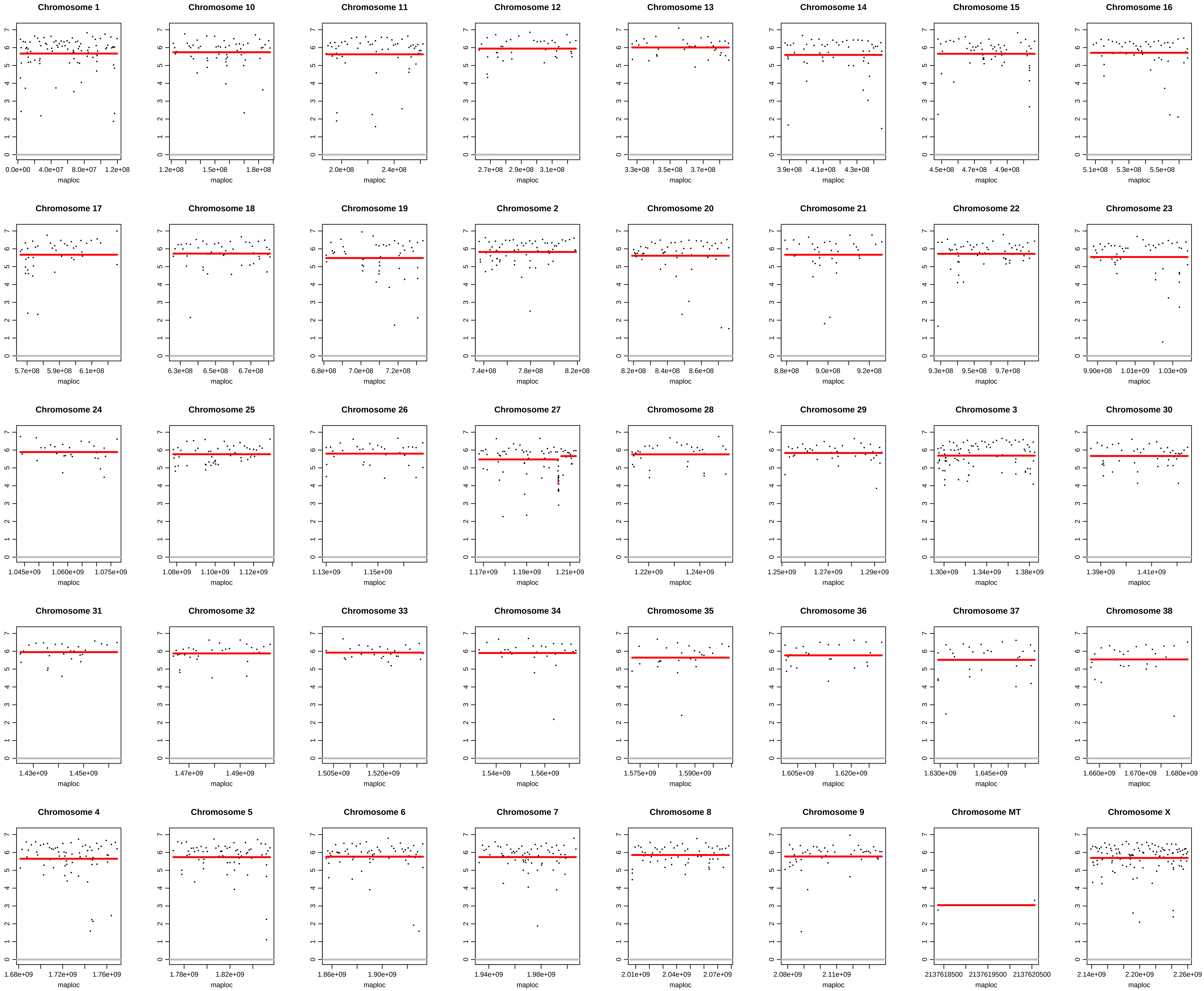

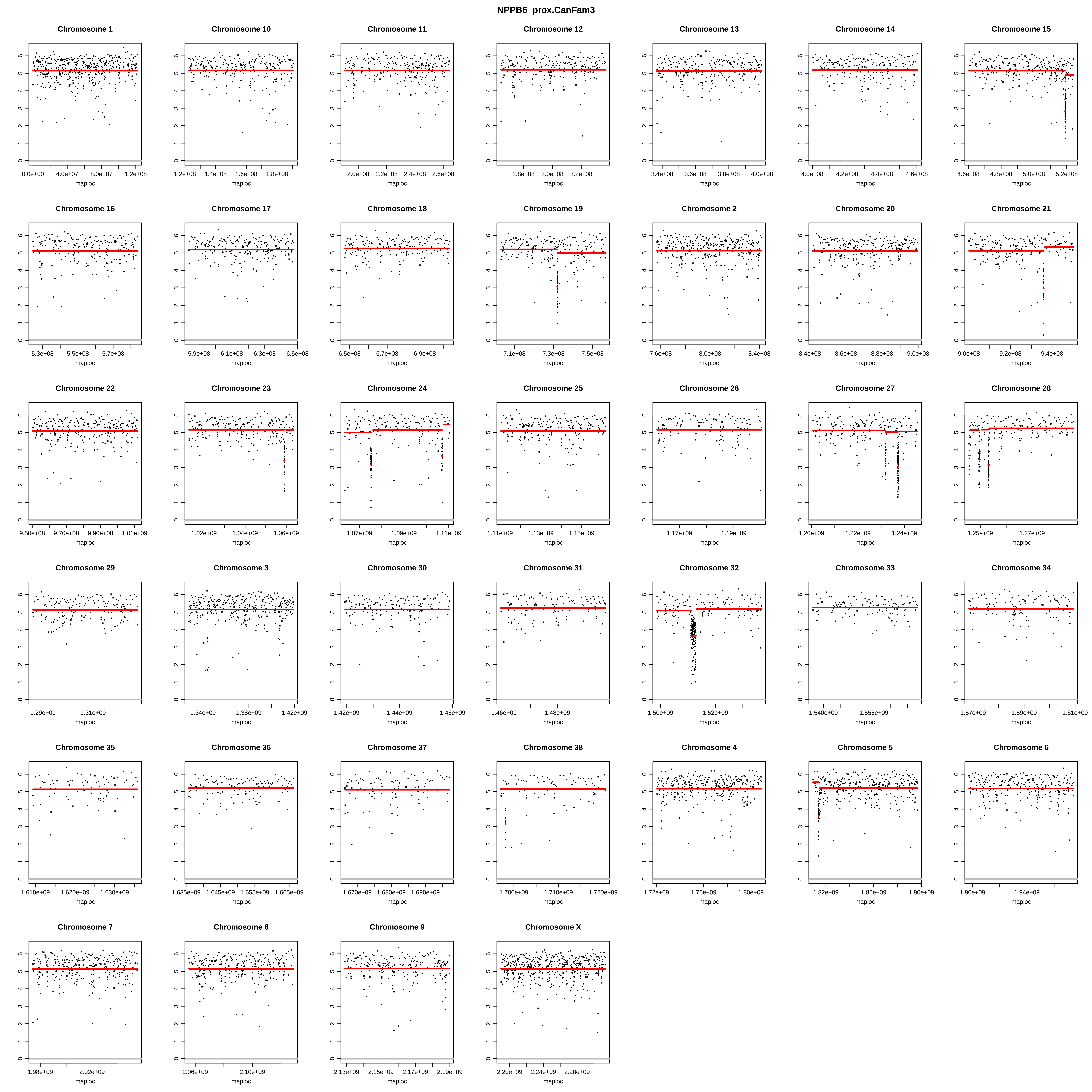
NPPB6_prox.CanFam3
Chromosome 1
Chromosome 10
Chromosome 11
Chromosome 12
Chromosome 13
Chromosome 14
Chromosome 15
Chromosome 16
Chromosome 17
Chromosome 18
Chromosome 19
Chromosome 2
Chromosome 20
Chromosome 21
Chromosome 22
Chromosome 23
Chromosome 24
Chromosome 25
Chromosome 26
Chromosome 27
Chromosome 28
Chromosome 29
Chromosome 3
Chromosome 30
Chromosome 31
Chromosome 32
Chromosome 33
Chromosome 34
Chromosome 35
Chromosome 36
Chromosome 37
Chromosome 38
Chromosome 4
Chromosome 5
Chromosome 6
Chromosome 7
Chromosome 8
Chromosome 9
Chromosome X
maploc

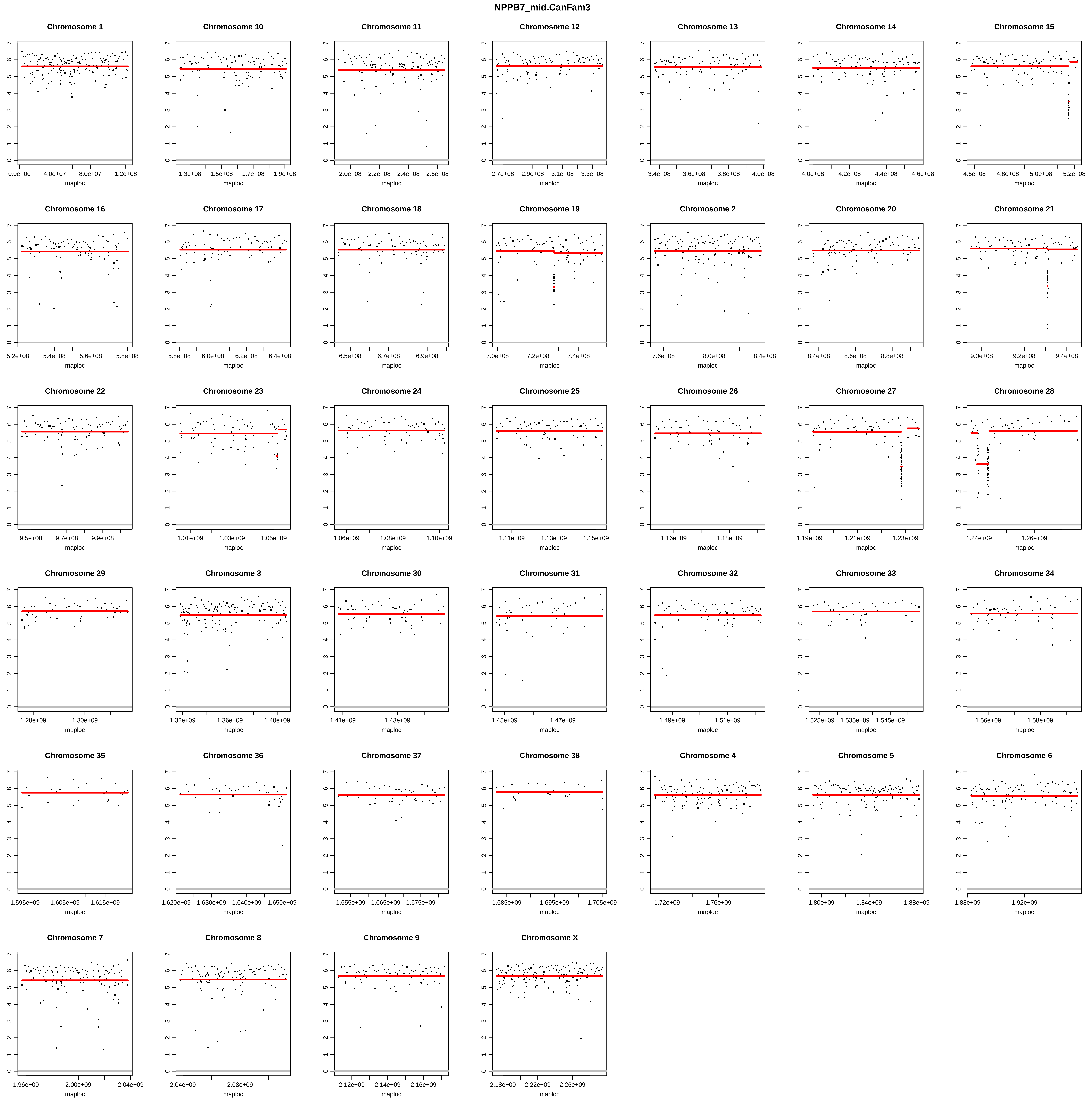
NPPB7_mid.CanFam3
Chromosome 1
Chromosome 10
Chromosome 11
Chromosome 12
Chromosome 13
Chromosome 14
Chromosome 15
Chromosome 16
Chromosome 17
Chromosome 18
Chromosome 19
Chromosome 2
Chromosome 20
Chromosome 21
Chromosome 22
Chromosome 23
Chromosome 24
Chromosome 25
Chromosome 26
Chromosome 27
Chromosome 28
Chromosome 29
Chromosome 3
Chromosome 30
Chromosome 31
Chromosome 32
Chromosome 33
Chromosome 34
Chromosome 35
Chromosome 36
Chromosome 37
Chromosome 38
Chromosome 4
Chromosome 5
Chromosome 6
Chromosome 7
Chromosome 8
Chromosome 9
Chromosome X
maploc

NPPB8_dist.CanFam3

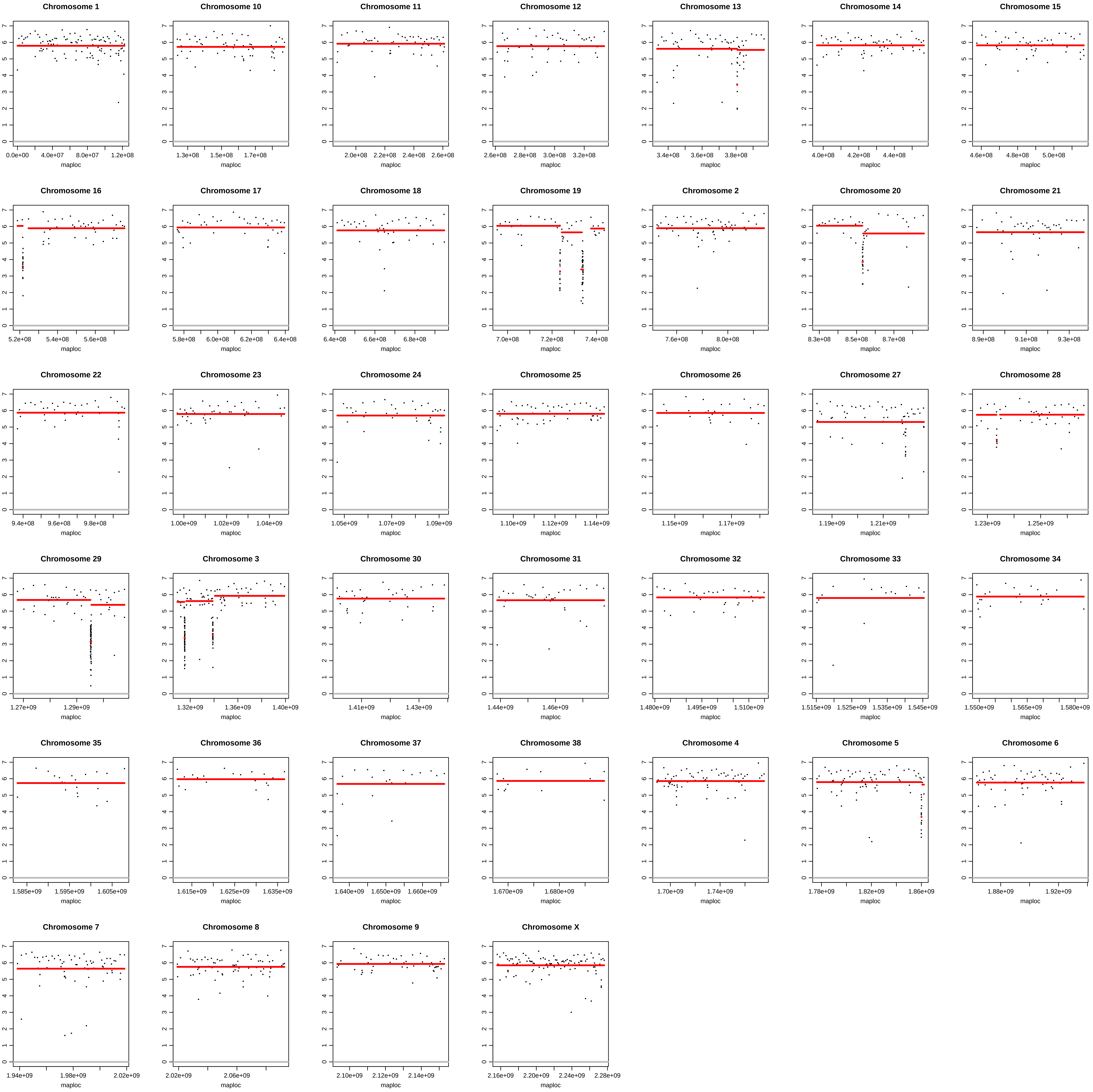

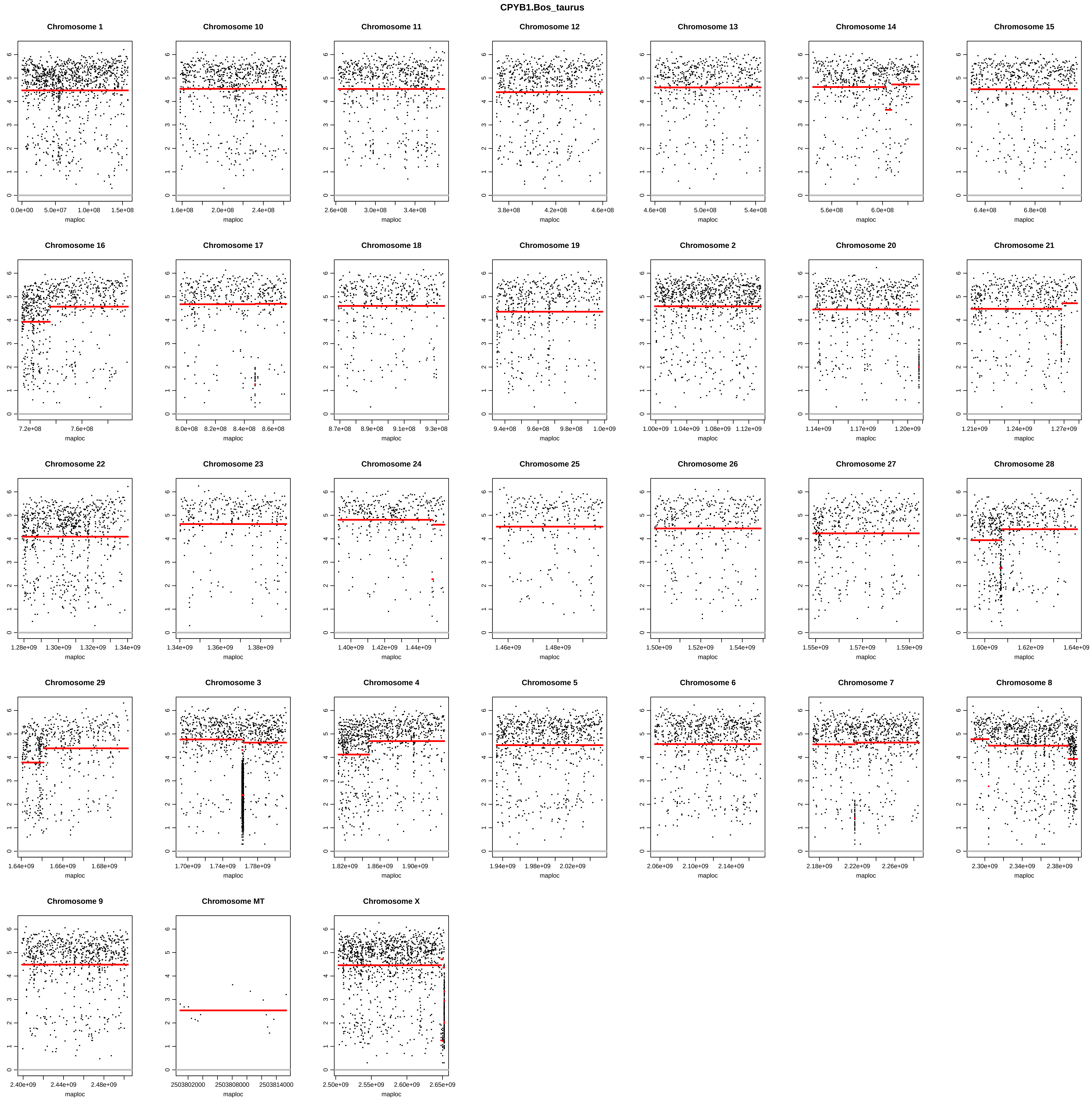
CPYB1.Bos_taurus
Chromosome 1
Chromosome 10
Chromosome 11
Chromosome 12
Chromosome 13
Chromosome 14
Chromosome 15
Chromosome 16
Chromosome 17
Chromosome 18
Chromosome 19
Chromosome 2
Chromosome 20
Chromosome 21
Chromosome 22
Chromosome 23
Chromosome 24
Chromosome 25
Chromosome 26
Chromosome 27
Chromosome 28
Chromosome 29
Chromosome 3
Chromosome 4
Chromosome 5
Chromosome 6
Chromosome 7
Chromosome 8
Chromosome 9
Chromosome MT
Chromosome X
maploc

CPYB2.Bos_taurus

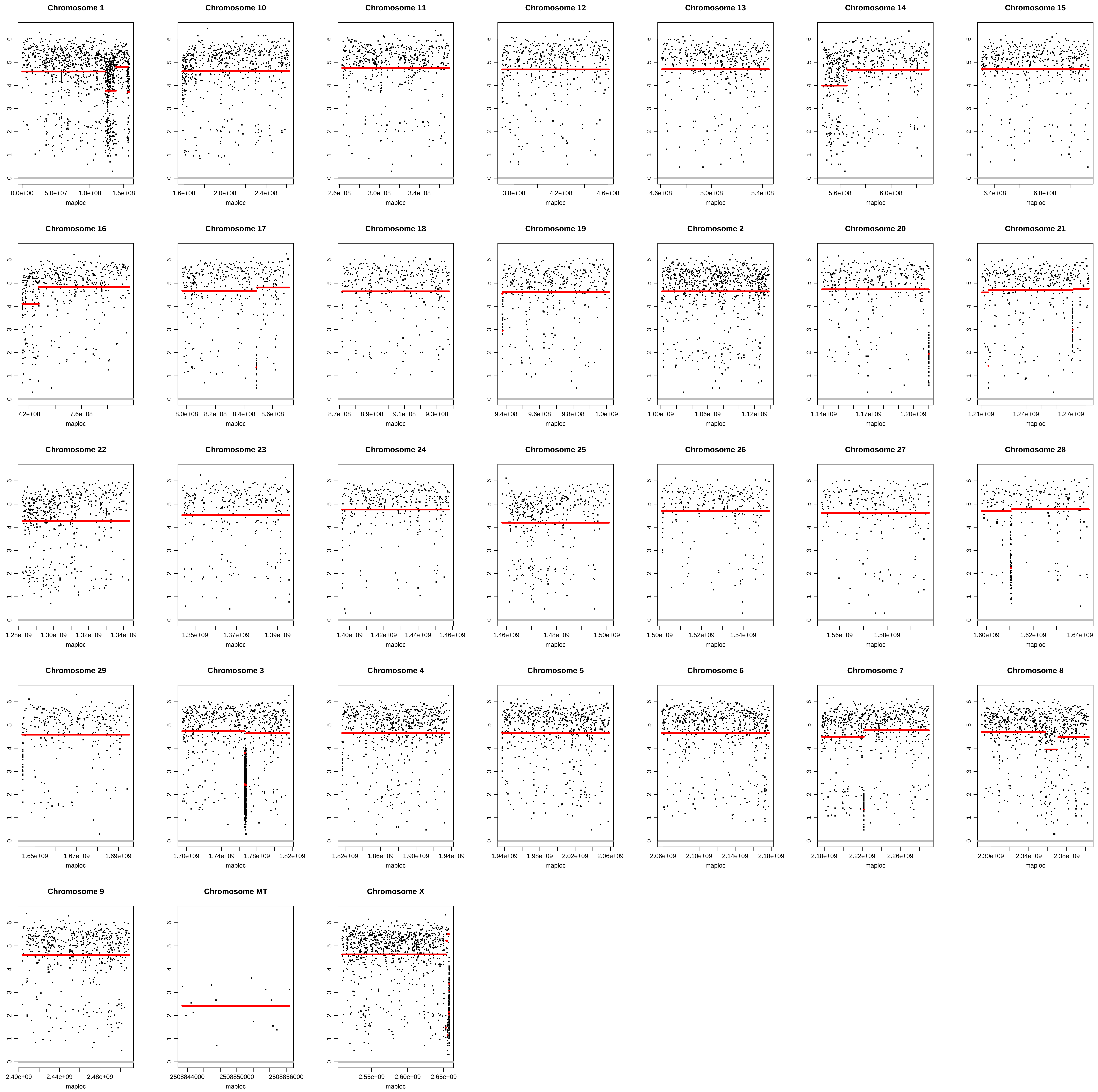

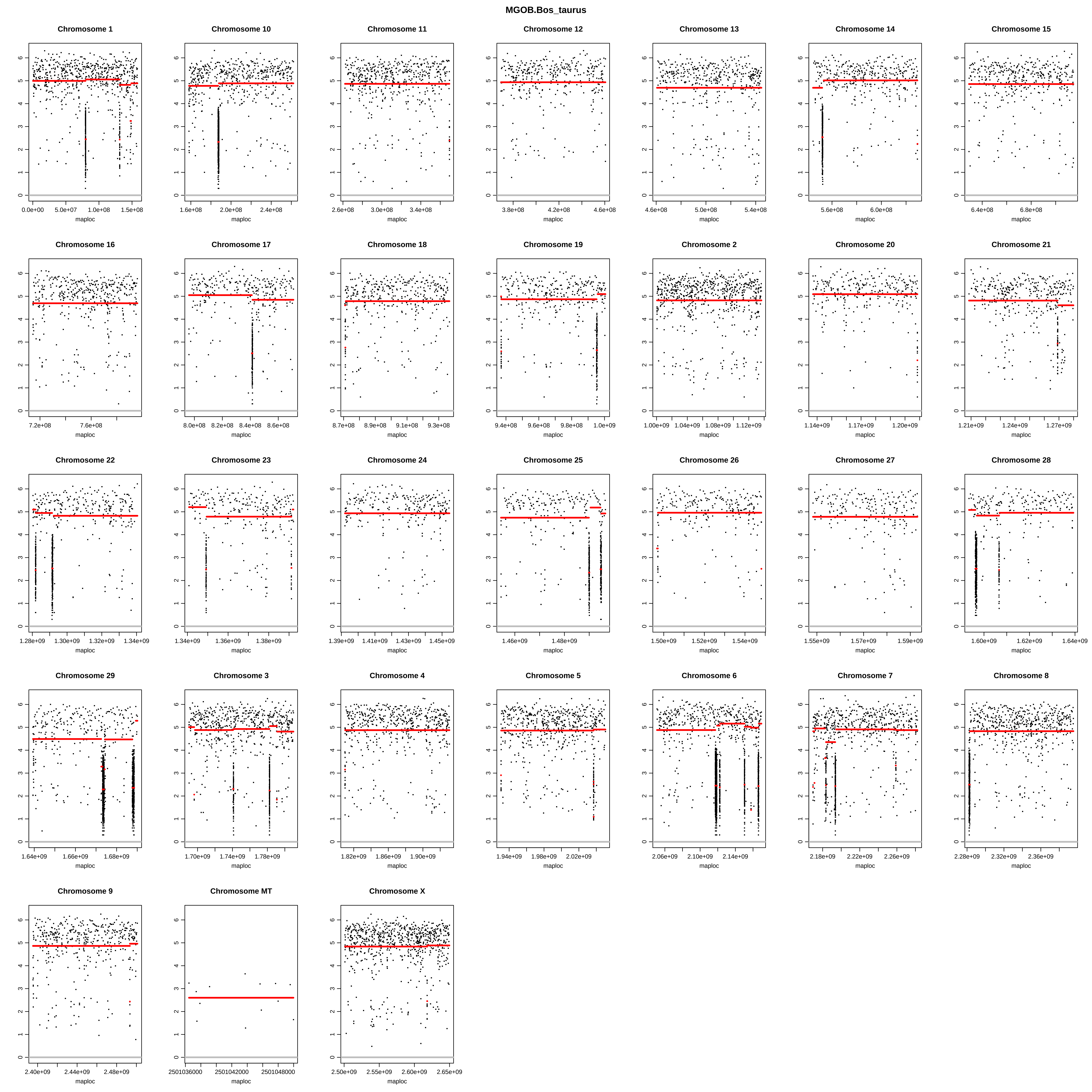

MGOB.Bos_taurus
Chromosome 1
0.0e+00 5.0e+07 1.0e+08 1.5e+08
Chromosome 10
1.6e+08 2.0e+08 2.4e+08
Chromosome 11
2.6e+08 3.0e+08 3.4e+08
Chromosome 12
3.8e+08 4.2e+08 4.6e+08
Chromosome 13
4.6e+08 5.0e+08 5.4e+08
Chromosome 14
5.6e+08 6.0e+08
Chromosome 15
6.4e+08 6.8e+08
Chromosome 16
7.2e+08 7.6e+08
Chromosome 17
8.0e+08 8.2e+08 8.4e+08 8.6e+08
Chromosome 18
8.7e+08 8.9e+08 9.1e+08 9.3e+08
Chromosome 19
9.4e+08 9.6e+08 9.8e+08 1.0e+09
Chromosome 2
1.00e+09 1.04e+09 1.08e+09 1.12e+09
Chromosome 20
1.14e+09 1.17e+09 1.20e+09
Chromosome 21
1.21e+09 1.24e+09 1.27e+09
Chromosome 22
1.28e+09 1.30e+09 1.32e+09 1.34e+09
Chromosome 23
1.34e+09 1.36e+09 1.38e+09
Chromosome 24
1.39e+09 1.41e+09 1.43e+09 1.45e+09
Chromosome 25
1.46e+09 1.48e+09
Chromosome 26
1.50e+09 1.52e+09 1.54e+09
Chromosome 27
1.55e+09 1.57e+09 1.59e+09
Chromosome 28
1.60e+09 1.62e+09 1.64e+09
Chromosome 29
1.64e+09 1.66e+09 1.68e+09
Chromosome 3
1.70e+09 1.74e+09 1.78e+09
Chromosome 4
1.82e+09 1.86e+09 1.90e+09
Chromosome 5
1.94e+09 1.98e+09 2.02e+09
Chromosome 6
2.06e+09 2.10e+09 2.14e+09
Chromosome 7
2.18e+09 2.22e+09 2.26e+09
Chromosome 8
2.28e+09 2.32e+09 2.36e+09
Chromosome 9
2.40e+09 2.44e+09 2.48e+09
Chromosome MT
2501036000 2501042000 2501048000
Chromosome X
2.50e+09 2.55e+09 2.60e+09 2.65e+09
0 1 2 3 4 5 6
maploc
